# The green peach aphid gut contains host plant microRNAs identified by comprehensive annotation of *Brassica oleracea* small RNA data

**DOI:** 10.1038/s41598-019-54488-1

**Published:** 2019-12-11

**Authors:** Max C. Thompson, Honglin Feng, Stefan Wuchty, Alex C. C. Wilson

**Affiliations:** 1 Department of Biology, University of Miami, Coral Gables, FL 33146 USA; 2 Present Address: Boyce Thompson Institute, 533 Tower Road, Ithaca, NY 14853 USA; 3 Department of Computer Science, University of Miami, Coral Gables, FL 33146 USA; 4 Center for Computational Science, University of Miami, Coral Gables, FL 33146 USA; 5 Sylvester Comprehensive Cancer Center, University of Miami, Miami, FL 33136 USA

**Keywords:** Plant sciences, Entomology, Coevolution

## Abstract

Like all organisms, aphids, plant sap-sucking insects that house a bacterial endosymbiont called *Buchnera*, are members of a species interaction network. Ecological interactions across such networks can result in phenotypic change in network members mediated by molecular signals, like microRNAs. Here, we interrogated small RNA data from the aphid, *Myzus persicae*, to determine the source of reads that did not map to the aphid or *Buchnera* genomes. Our analysis revealed that the pattern was largely explained by reads that mapped to the host plant, *Brassica oleracea*, and a facultative symbiont, *Regiella*. To start elucidating the function of plant small RNA in aphid gut, we annotated 213 unique *B. oleracea* miRNAs; 32/213 were present in aphid gut as mature and star miRNAs. Next, we predicted targets in the *B. oleracea* and *M. persicae* genomes for these 32 plant miRNAs. We found that plant targets were enriched for genes associated with transcription, while the distribution of targets in the aphid genome was similar to the functional distribution of all genes in the aphid genome. We discuss the potential of plant miRNAs to regulate aphid gene expression and the mechanisms involved in processing, export and uptake of plant miRNAs by aphids.

## Introduction

**Figure 1:**
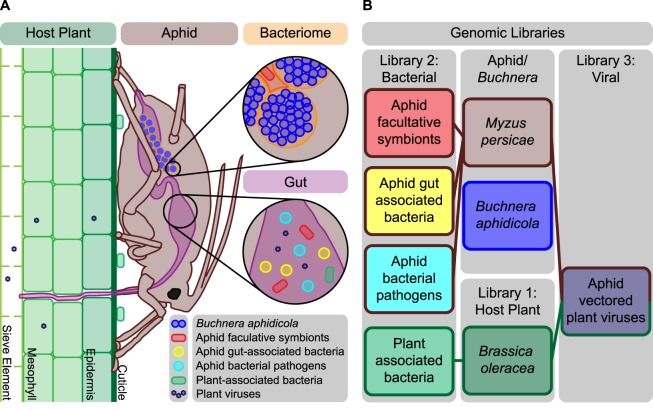
Aphid bacteriome and gut tissues were mapped against aphid, *Buchnera*, and host plant genomes as well as a bacterial library and viral library. (**A**) *Myzus persicae* feeding on *Brassica oleracea* with the aphid stylet passing through the cuticle, epidermis, and mesophyll to reach the sieve element and extract phloem. Aphid gut is colonized by a microbiome that includes facultative symbionts, gut-associated bacteria, bacterial pathogens, plant associated bacteria and viruses. The bacteriome is the aphid organ made up of bacteriocyte cells that house *Buchnera* and sheath cells that sometimes are infected with facultative symbionts. (**B**) Custom libraries: (1) Host plant, (2) Bacterial and (3) Viral.

Most insects in the order Hemiptera are herbivores that feed on plant sap^1^. Typically, insects that feed on plant sap critically depend on bacteriome housed endosymbionts to supplement dietary shortfalls in essential amino acids and vitamins^2–4^. Many insects also host facultative symbionts that confer benefits such as resistance to attack by parasitoids and increased tolerance to heat stress^5^. For example, the pea aphid, *Acyrthosiphon pisum*, that obligately hosts the endosymbiont *Buchnera aphidicola*, has also been reported to host a facultative symbiont, *Candidatus* Hamiltonella defensa encoding a bacteriophage that provides strong protection against parasitic wasps^6,7^. While aphid guts contain a relatively limited microbiome^8,9^, aphids such as *M. persicae*, vector hundreds of different plant viruses^10,11^. Thus, the species interaction network of an aphid includes its obligate and facultative symbionts, its host plant, its gut microbiome, the microbiome of its host plant, and the viruses it vectors (Fig. 1A).

**Figure 2:**
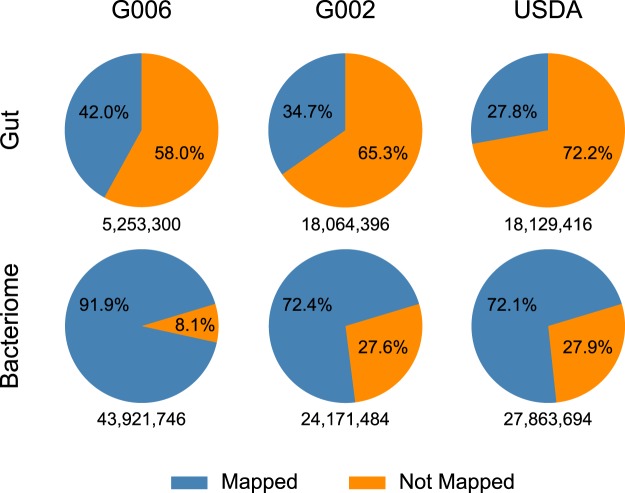
More than 58% of small RNA reads from aphid gut do not map to the aphid or endosymbiont genomes. In contrast, less than 28% of small RNA reads from aphid bacteriomes do not map to the aphid or endosymbiont genome. Pie charts show the proportion of gut or bacteriome reads that aligned to the aphid and/or *Buchnera* genomes with less than two mismatches. Data are shown for three *M. persicae* lines: G006, G002, and USDA. Numbers below the pie charts refer to the total number of reads in the respective filtered dataset.

In this paper, we study small RNA datasets from *M. persicae*. Preliminary data processing of the *M. persicae* small RNA data available in the National Center for Biotechnology Information (NCBI) data repository as BioProject PRJNA395356 identified a striking difference between gut and bacteriome tissue samples (see Fig. 1A for arrangement of tissues in aphid host) in the proportion of total reads that mapped to the *M. persicae* and *Buchnera* genomes (Fig. 2). This difference, which was not explored by Feng *et al*.^12^, motivated the current work.

We set out to determine the source of unmapped small RNA reads in *M. persicae* gut samples, by mapping these reads against three genomic libraries. The first contained the genome of cabbage, *Brassica oleracea*^13^, since our aphid lines were raised on *B. oleracea* seedlings. The second contained genomes of both beneficial and pathogenic bacteria, that are known to be associated with aphids^8,14^, while the third contained genomes of aphid vectored plant viruses^15,16^ (Fig. 1B). Our goal was to leverage all available genomic information for members of the *M. persicae* species interaction network to determine the source of small reads that did not map to the *M. persicae* or *Buchnera* genomes.

Surprisingly, we found that upwards of 21% of small RNA reads in aphid gut samples mapped to the host plant genome. Motivated by recent demonstration of miRNA function across kingdoms^17^, we next asked if the *B. oleracea* small read data included any miRNAs. To address this question, we leveraged three existing *B. oleracea* small RNA datasets to annotate *B. oleracea* miRNAs using the current approaches recommended by Axtell & Meyers^18^. Following annotation, we found evidence of 32 *B. oleracea* miRNAs in aphid gut samples. Intrigued by the possibility that these *B. oleracea* miRNAs regulate aphid gene expression, we predicted targets in both the *B. oleracea* and *M. persicae* genomes. We found that the distribution of targets across the *M. persicae* genome were dissimilar to that observed in the *B. oleracea* genome targets that were strongly enriched for functions associated with transcription.

## Results

### More than one fifth of small RNA reads from aphid gut tissue samples map to the host plant genome

**Figure 3:**
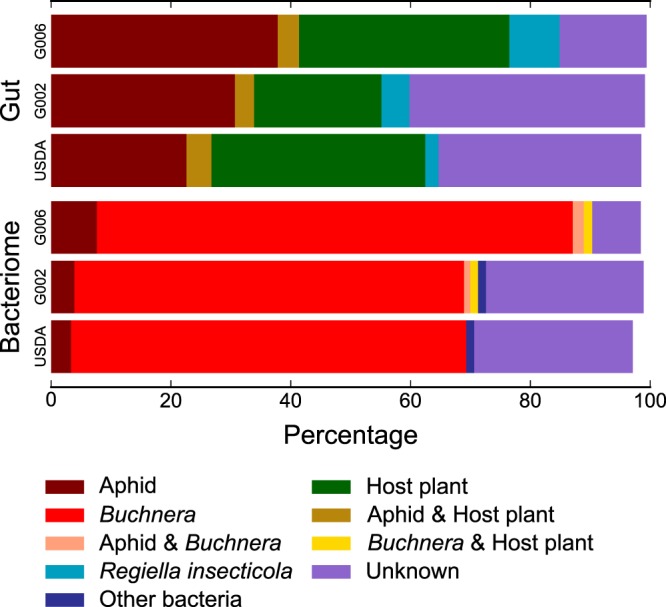
More than 21% of small RNA reads in aphid gut map exclusively to the genome of the host plant, *B. oleracea* (green). Bars show the proportion of reads that aligned to the aphid, *Buchnera*, and host plant genomes for gut and bacteriome tissues in three *M. persicae* lines: G006, G002, and USDA. Categories <1% not displayed (see Table S1).

We analyzed *M. persicae* bacteriome and gut tissue small RNA data from three genetically discrete aphid lines. In bacteriomes, 78.8% ± 11.3% of small RNA reads mapped to the aphid and/or *Buchnera* genome. In contrast, in gut samples, only 34.8% ± 7.1% of reads mapped to the aphid and/or *Buchnera* genome (Fig. 2). Remarkably, in gut samples, 34.4% ± 8.6% of small RNA reads mapped to the host plant, *B. oleracea*, with 30.8% ± 8.2% mapping only to *B. oleracea* (Fig. 3, Supplementary Table S1).

### Small RNA reads from aphid gut tissue are of limited bacterial origin and not of viral origin

For gut samples, we mapped reads of unknown origin against two microbial libraries. The first was a bacterial library that contained the genomes of aphid facultative symbionts, aphid gut associated bacteria, and plant associated bacteria, while the second was a viral library that contained the genomes of plant viruses that aphids are known to vector (Fig. 1B, Supplementary Table S2). We found that between 2.5% and 8.6% of all small RNA reads from aphid gut tissues mapped exclusively to the bacterial library while, at most only 0.004% of all small RNA reads mapped exclusively to the viral library (Supplementary Tables S1 and S2). Most of the bacterial library mapping was attributed to *Regiella insecticola* with at most only 0.5% of the gut small RNA reads mapping to additional bacteria (that were not *Buchnera*).

### Plant miRNAs are present in aphid gut and bacteriome tissue

To test our hypothesis that *B. oleracea* small RNAs found in *M. persicae* gut contain plant miRNAs, we annotated miRNAs in the *B. oleracea* genome using publicly available small RNA-seq data. Out of a total of 147 miRNA precursors in the *B. oleracea* genome 138 were homologous to known plant miRNA precursors (miRBase v22) and 9 were novel miRNA precursors (Supplementary Table S3). The 147 precursors encoded 213 miRNAs, a set that includes unique miRNAs isolated from both the 5′ and 3′ ends of the miRNA precursors. We next asked if any of these 213 miRNAs were present in our aphid gut and bacteriome datasets. In gut samples, we found perfect matches for 32 *B. oleracea* miRNAs that were each represented between one and 370 times (Supplementary Table S4). Seven of the 32 *B. oleracea* miRNAs were represented in the guts of all three *M. persicae* lines. These seven miRNAs map to 13 different miRNA precursors (because some miRNAs are encoded by duplicated precursors), including bol-miR395d, bol-miR_novel_104, and bol-miR168b-3p, as well as three members of the bol-miR166 family (166a-3p, 166b-3p, and 166e-3p), three members of the bol-miR159 family (159, 159a, 159c), two members of the bol-miR162 family (162-5p, 162a-5p), and two members of the bol-miR168 family (168a, 168c). In contrast, we found perfect matches for only four *B. oleracea* miRNAs in the aphid bacteriome tissue datasets with each miRNA represented between one and 16 times. Two of the four *B. oleracea* miRNAs identified in bacteriome were also represented in the gut datasets (Supplementary Table S4) that mapped to four precursors: bol-miR156-5p and three members of the bol-miR166 family (166a-3p, 166b-3p, and 166e-3p).

### Plant miRNAs present in aphid gut have target sites in aphid and plant transcriptomes

**Figure 4:**
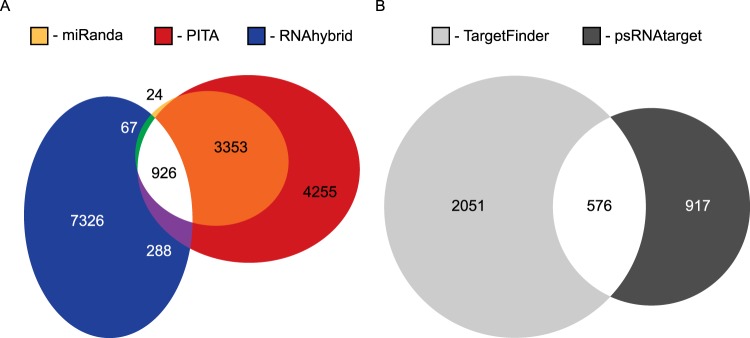
Prediction of targets of plant miRNAs identified 926 and 576 putative targets in the aphid and plant genomes, respectively. (**A**) Proportional Venn diagram of targets in aphid 3′-UTRs predicted by miRanda^52^, PITA^53^ and RNAhybrid^54^. (**B**) Proportional Venn diagram of targets in plant genome predicted by TargetFinder^55^ and psRNAtarget^51^. Proportional Venn diagrams were produced using euler*APE* v3.0.0^61^.

Using the intersection of three miRNA targeting programs specific to animal genomes (miRanda, PITA and RNAhybrid), we predicted 926 *M. persicae* 3′ UTRs as putative targets for the 32 *B. oleracea* miRNAs detected in *M. persicae* gut (Fig. 4). The 926 predicted 3′ UTR targets associated with 965 *M. persicae* mRNA isoforms. In contrast, in the *B. oleracea* genome, using the intersection of two miRNA target prediction programs specific to plant genomes (psRNAtarget and TargetFinder), we predicted 576 *B. oleracea* target sites for the 32 *B. oleracea* miRNAs detected in *M. persicae* gut (Fig. 4).

### Targets of plant miRNAs present in aphid gut have organism-specific COG profiles

We performed a broad functional analysis by assigning the transcripts targeted by the 32 *B. oleracea* miRNAs to clusters of orthologous groups (COGs)^19^. We used Monte Carlo simulations to test the significance of over and under-representation of each COG in our predicted target sets relative to the representation of COGs in the official gene set of the corresponding genome. In the *M. persicae* genome, we assigned a functional COG to 664 of the 965 predicted targets, and in the *B. oleracea* genome, we assigned functional COGs to 330 of the 576 predicted targets.

**Figure 5:**
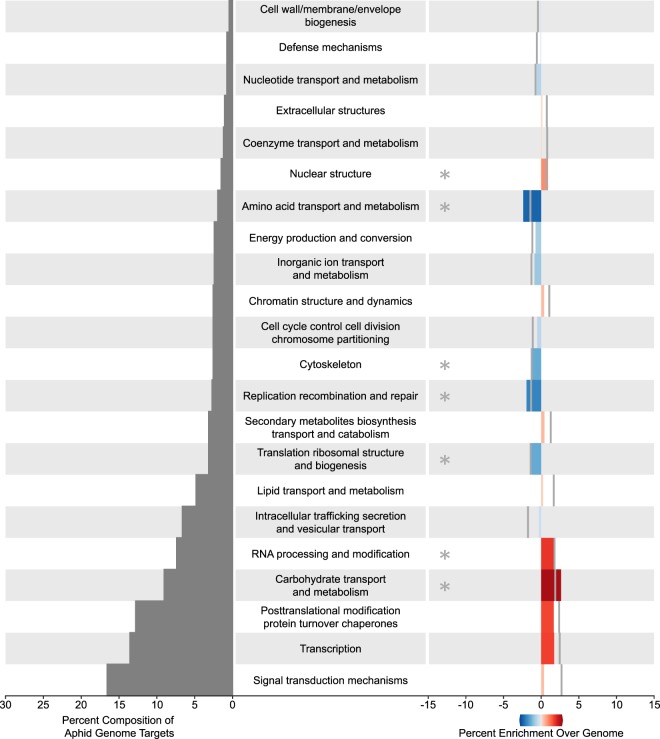
Cluster of Orthologous Group (COG) distribution of altered plant miRNA targets over aphid genome. (Left) Percentage of targets found in each COG in increasing order. (Right) Percent difference between the distribution of COGs in the targets of the 32 *B. oleracea* miRNAs in the aphid genome and of the whole aphid genome. Significant categories are marked with an asterisk (*p* < 0.05). On the right, grey bars mark the value needed to achieve signifcant overrepresentation (positive % enrichment) or underrepresentation (negative % enrichment).

For the set of targets in the aphid genome, three COGs, “Carbohydrate transport and metabolism” (*p* = 3.5 × 10^−3^), “RNA processing and modification” (*p* = 2.2 × 10^−2^), and “Nuclear structure” (*p* = 6.2 × 10^−3^) were significantly overrepresented while four COGs “Amino acid transport and metabolism” (*p* = 2.0 × 10^−4^), “Cytoskeleton” (*p* = 1.6 × 10^−2^), “Replication, recombination, and repair” (*p* = 1.5 × 10^−3^), and “Translation, ribosomal structure and biogenesis” (*p* = 2.6 × 10^−2^) were significantly underrepresented (Fig. 5). Notably, the categories over and under-represented in the *M. persicae* genome targets differed from those over and under-represented in the *B. oleracea* genome.

**Figure 6:**
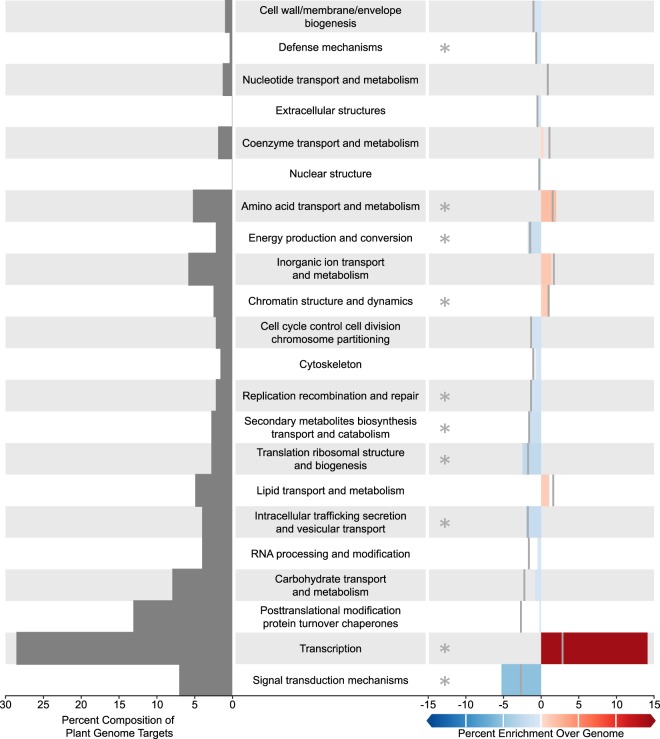
Cluster of Orthologous Group (COG) distribution of altered plant miRNA target over plant genome. (Left) Percentage of targets found in each COG in increasing order. (Right) Percent difference between the distribution of COGs in the targets of the 32 *B. oleracea* miRNAs in the plant genome and of the whole plant genome. Significant categories are marked with an asterisk (*p* < 0.05). On the right, grey bars mark the value needed to achieve signifcant overrepresentation (positive % enrichment) or underrepresentation (negative % enrichment).

In the set of targets in the *B. oleracea* genome, three COGs “Transcription” (*p* < 1 × 10^−6^), “Amino acid transport and metabolism” (*p* = 6.0 × 10^−3^), and “Chromatin structure and dynamics” (*p* = 1.8 × 10^−2^) were significantly overrepresented with “Transcription” being the most significantly overrepresented as well as the COG that included the largest fraction of the *B. oleracea* genome targets (28%, Fig. 6). Interestingly, “Amino acid transport and metabolism” was significantly overrepresented in the *B. oleracea* genome targets while it was underrepresented in the *M. persicae* genome targets. The *B. oleracea* genome targets also included seven COGs that were significantly underrepresented: “Signal transduction mechanisms” (*p* = 2.5 × 10^−5^), “Intracellular trafficking, secretion, and vesicular transport” (*p* = 1.5 × 10^−2^), “Translation, ribosomal structure and biogenesis” (*p* = 1.6 × 10^−3^), “Secondary metabolites biosynthesis and catabolism” (*p* = 1.9 × 10^−2^), “Energy production and conservation” (*p* = 1.1 × 10^−2^), “Replication, recombination, and repair” (*p* = 4.2 × 10^−2^), and “Defense mechanisms” (*p* = 2.1 × 10^−2^). In both genomes, “Replication, recombination, and repair” was significantly underrepresented.

### Plant-derived, aphid gut small RNAs 17–35 nucleotides in length that map to plant miRNA precursors are almost exclusively miRNAs

**Figure 7:**
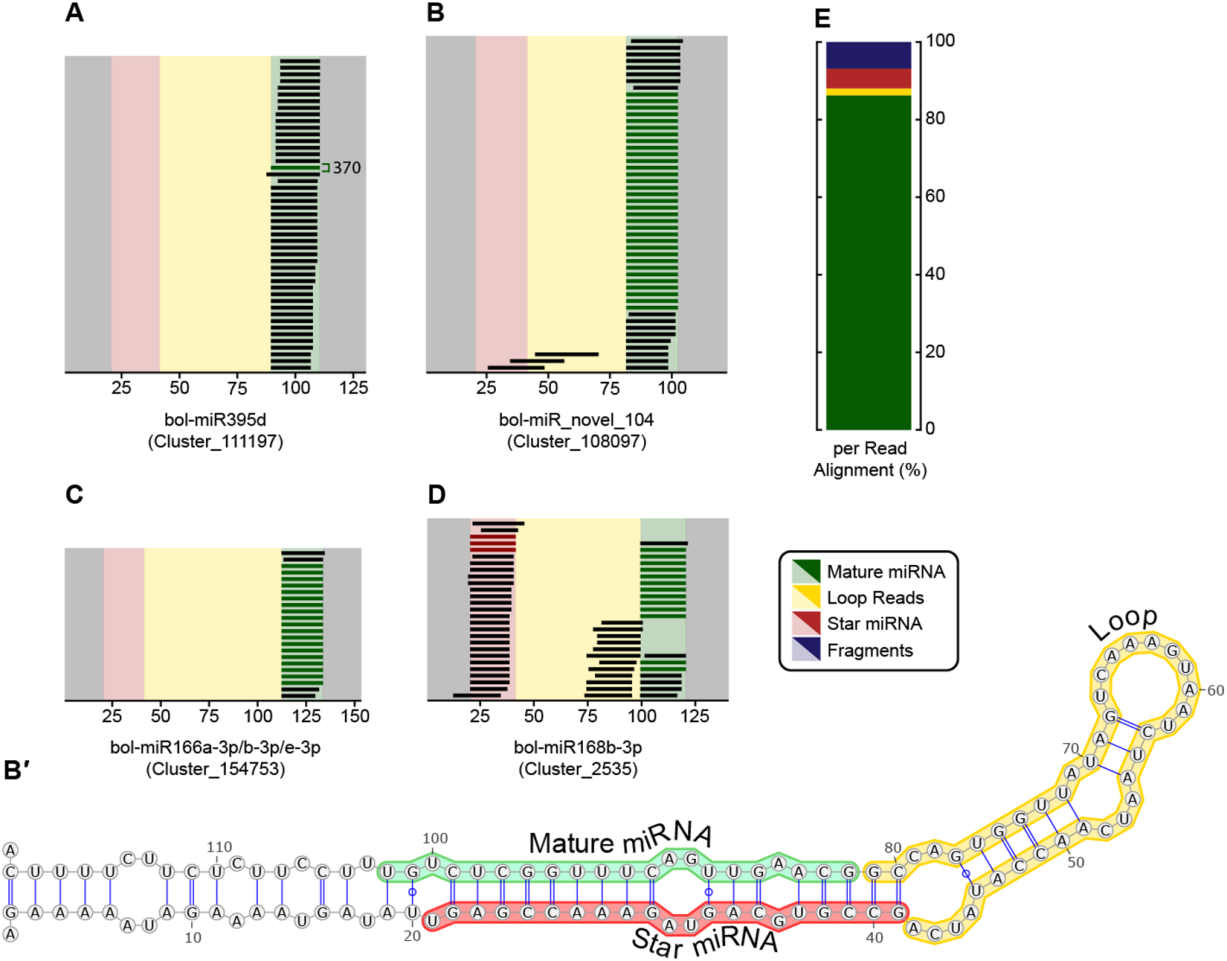
Alignment of plant small RNA data against plant miRNA precursors. (**A–D**) Representation of *M. persicae* gut small RNA reads that exactly map to *B. oleracea* miRNA precursors. While red reads are perfect star miRNA matches green reads are perfect mature miRNA matches. X-axis indicates precursor nucleotide position, miRNA name and precursor identity in parentheses. (**A**) One mature miRNA sequence is shown in place of the 370 that were aligned in that position. (**B′**) The secondary structure of the precursor displayed in B. (**E**) Categorization of all reads aligned to plant miRNA precursors across all gut datasets. Reads are assigned to the mature miRNA, star miRNA, and loop sections by aligning with a maximum of one nucleotide overlap with adjacent sections. Any read that overlapped two sections by more than one nucleotide was classified as a fragment.

We aligned our size-selected and filtered *M. persicae* gut small RNA data against full-length miRNA precursors of the 32 *B. oleracea* miRNAs present in our *M. persicae* gut datasets (Supplementary Document S1). Precursor miRNAs comprise 5′ and 3′ end regions, mature and star miRNAs, and a loop region (Fig. 7B’). None of the small RNA reads in our gut dataset aligned to 5′ or 3′ end regions while 1.8% aligned to loop regions and 91.4% of reads aligned perfectly to mature or star miRNA sequences (Fig. 7). The remaining 6.8% of reads that aligned to the 32 miRNA precursors were fragments that spanned two regions within a miRNA precursor *e.g*. fragments that spanned a star miRNA and a loop region (Fig. 7B), or a 5′ end fragment and a star miRNA (Fig. 7D).

## Discussion

We interrogated the striking difference between the proportion of total small RNA reads that mapped to the *M. persicae* and *Buchnera* genomes in *M. persicae* gut versus bacteriome tissues (Fig. 2). Our analyses revealed that two members of the *M. persicae* species interaction network, the host plant, *B. oleracea*, and an aphid facultative symbiont, *Regiella insecticola*, were the source of a large fraction of the unmapped small RNA reads in *M. persicae* gut libraries (Supplementary Table S1). In all three *M. persicae* lines, the host plant accounted for the plurality of unmapped reads while *Regiella* was the source of a small, yet substantial fraction of the unmapped reads in all three lines (Supplementary Table S1). While we are not the first to detect plant (diet) derived small RNA in aphids, our work is, to the best of our knowledge, the first to document that the abundance of plant small RNA is strikingly different among aphid tissues^20,21^. This is also not the first study to document the presence of *Regiella* in *M. persica*e as *Regiella* infection was previously reported in a single *M. persicae* line from Australia^22^. However, detection of *Regiella* in all three of the *M. persicae* lines used in this study was surprising as the presence of *Regiella* infection had not been revealed by earlier genome sequencing projects^23,24^. Earlier failure to detect *Regiella* infection coincident with previous work sequencing the genomes of *Buchnera* from *M. persicae* lines USDA and G002 likely resulted from the filtration methods used to enrich for *Buchnera* in genomic DNA preparations^5,24^. While failure to detect *Regiella* infection coincident with sequencing the G006 *M. persicae* genome is less clear, it likely resulted from low *Regiella* titre and bioinformatic filtering of bacterial contaminants^23^.

Following identification of *B. oleracea* small RNAs in our *M. persicae* gut libraries, we next asked if the *B. oleracea* small read data included any miRNAs. While previous studies reported annotation of 169^25^ (from a diversity of tissues), 159^26^ (from mature leaves), and 156 (from young leaves)^27^ miRNAs in *B. oleracea*, the intersection of these three sets only indicated 15 miRNAs (Supplemental Fig. S2). Further, in March 2018, the reference miRNA database, miRBase v22, contained only 10 *B. oleracea* unique miRNAs. Thus, we performed a new annotation of miRNAs in *B. oleracea* that leveraged all available datasets and followed the newly updated criteria for identification of plant miRNAs from big data RNAseq projects^18^. We predicted 147 miRNA precursors that produced 213 unique miRNAs (note that miRNAs can be encoded at both the 5′ and 3′ end of precursors, and two different precursors can encode identical miRNAs). Of the 213 miRNAs, 86 were previously reported in at least one of the earlier *B. oleracea* annotations^25–27^ (Supplemental Fig. S2), and out of the 213, 203 are members of known miRNA families (miRBase v22). The remaining 10 are novel miRNAs that lack homology to known miRNA families in miRBase v22. However, three of these 10 were previously reported by Zhang *et al*.^27^, and two were identified previously in the genus *Brassica* by Wei *et al*.^28^. The remaining five novel miRNAs are reported for the first time in this study (Supplementary Table S3).

Leveraging our new annotation of *B. oleracea* miRNAs we identified 32 *B. oleracea* miRNAs in our *M. persicae* gut data. While plant xylem sap is devoid of RNA^29^, plant phloem sap contains large numbers of small RNA molecules that generate a small RNA profile distinct from other plant tissues^29–32^. Out of the 32 *B. oleracea* miRNAs that we found in *M. persicae* gut, 16 have been found previously in phloem sap^29–32^, including 12 identified from *Brassica napus* phloem^29,30^ (Supplementary Table S4). Further, all four of the miRNAs found in bacteriome tissue were previously identified in *B. napus* phloem^30^. Overall, our results suggest that host plant miRNAs found in aphid tissues are members of the *B. oleracea* circulating miRNA profile that facilitates the systemic spread of post-transcriptional gene regulation.

An outstanding question at this time concerns function. Are the *B. oleracea* circulating miRNAs that we found in *M. persicae* gut functional? Do these *B. oleracea* miRNAs have targets in the *M. persicae* genome? Do they regulate *M. persicae* gene expression, or are they simply food? The ease with which it is possible to generate large amounts of small RNA and mRNA data has resulted in a recent flood of studies claiming regulation of gene expression in the genome of an organism by miRNAs encoded in the genome of its food. Unfortunately, in many cases authors have been quick to assume function in the absence of experimental validation^33–36^. Here, we predicted mRNA targets in the *B. oleracea* and *M. persicae* genomes for the 32 *B. oleracea* miRNAs found in *M. persicae* gut. Our analysis of the *M. persicae* genome failed to identify signatures of enrichment in 19 of the 22 COGs assigned to predicted targets relative to the COG composition of all genes in the *M. persicae* genome. That said, three *M. persicae* COGs were significantly enriched in targets relative to the *M. persicae* genome (Fig. 5); however, the degree of enrichment of these three COGs compared to the degree of enrichment of *B. oleracea* targets in the COG “Transcription” relative to the *B. oleracea* genome (Fig. 6) is much lower. While these broad-scale COG analyses revealed a lack of directed evolution in plant miRNAs targeting aphids, they also say little about the ability of individual plant miRNAs to elicit a physiological effect in aphids. However, experimental evidence from transgenic plants is compelling in arguing for the potential of *B. oleracea* encoded miRNAs to elicit a biological effect in *M. persicae*. We emphasize this point because aphids fed on transgenic plants expressing artificial miRNAs and dsRNA that target individual genes in the aphid genome, show reduced expression of the targeted gene^37–40^. Additionally, the cotton bollworm and the Colorado potato beetle have been shown to accumulate plant produced small RNA fragments and a corresponding reduction in expression of the targeted genes^41,42^. Thus, such plant-mediated RNAi experiments serve as evidence that host plant genome encoded miRNAs could indeed regulate aphid gene expression *in vivo*.

If plant circulating miRNAs do indeed function to target aphid gene expression, what processing, export, transport and uptake mechanisms may be involved? By integrating our observations with current knowledge about the production, processing, and export of miRNAs, we propose that trafficking of plant miRNA into aphid tissues is mediated by exosomes. First, *B. oleracea* miRNA precursors are processed yielding mature and star miRNAs within the plant cells^43^. Next, miRNAs and proteins that will be secreted from the plant cells are selectively loaded with *B. oleracea* Argonaute 1 (Ago1)^44^ into exosomes within multivesicular bodies^43^. Co-packing of the miRNAs with Ago1 facilitates stabilization and function of the secreted miRNAs when the exosome reaches its destination^45^. As the multivesicular bodies fuse with the plant cell membrane, the miRNA-containing exosomes are exocytosed into the circulating apoplastic fluid of *B. oleracea*^43,46,47^. The apoplast then carries the exosomes to the phloem where they can achieve long-distance transport and contact with the feeding aphids. Since aphids feed passively on moving phloem, miRNA-containing exosomes are brought into the aphid digestive tract through the aphid stylet when it is inserted into the plant sieve elements. Once in the aphid digestive tract, the host plant miRNAs are protected by their exosome membrane and move into aphid gut cells by endocytosis^44^. Once inside aphid cells, the *B. oleracea* miRNAs and associated proteins are released to function alongside the aphid’s own long-distance miRNAs^37–39,48^.

Here we document the presence of *B. oleracea* circulating miRNAs in gut and bacteriome tissues of the aphid *M. persicae*. COG analyses did not reveal strong targeting of any one functional group of aphid genes by host plant miRNAs. However, we argue that other work with transgenic plants that have been engineered to intracellularly express miRNAs and dsRNA that target aphid genes demonstrate the presence of a physiological mechanism for cross-kingdom miRNA targeting from plants to aphids. Future *in vitro* and *in vivo* studies are required to test the cross-kingdom miRNA::mRNA interactions predicted here.

## Methods

We used the small RNA (sRNA) data for *Myzus persicae* lines G006, G002 and USDA from NCBI BioProject PRJNA395356. Sample preparation and initial data processing is described in Feng *et al*.^12^. Briefly, we processed the raw small RNA sequencing reads by first filtering out low quality reads, and then trimming sequencing adapters and primers. Mature miRNAs are typically 22 nucleotides in length, and thus following initial filtering and trimming, we retained reads that were 17–35 nucleotides long. For each sample, the set of 17–35 nucleotide long processed reads were used for genome mapping as described below. Aphid lines were raised at the University of Miami on *Brassica oleracea* var. Wisconsin Golden Acre.

### Identification of source genome

Using Bowtie2 v2.2.6. (parameters: -L 10–very-sensitive -a) gut and bacteriome sRNA data were each aligned against the *M. persicae* G006 v2.0 genome^23^ from AphidBase (http://www.aphidbase.com) and their respective *Buchnera aphidicola* genomes: G006, NCBI RefSeq GCF_001939165.1^23^; G002, NCBI RefSeq GCF_000521565.1^24^; and USDA, NCBI RefSeq GCF_000521525.1^24^. Following alignment, we identified reads that aligned with less than two mismatches and those that failed to align to the aphid and/or *Buchnera* genome; these reads were interrogated for alignment against three custom libraries. The first library contained the genome of the host plant, *Brassica oleracea*. The *B. oleracea* var. oleracea genome (NCBI RefSeq: GCF_000695525.1^13^) was used because the genome of var. Wisconsin Golden Acre had not been sequenced. The second library contained the genomes of aphid secondary bacterial symbionts, aphid gut associated bacteria, and plant associated bacteria^14^ (Supplementary Table S2). The third library contained the genomes of plant viruses that aphids have been shown to vector^15,16^ (Supplementary Table S2). Genomes for bacteria and viruses were collected from the NCBI Assembly database. For each species or genus, preference was given to genomes with reference status and then representative status in the NCBI Assembly database. If those were not available, a complete genome was utilized. If nothing met the above criteria, manual searches were performed, using the most complete version of the genome. When only genus information was available, all species under the genus having at least a complete genome were utilized.

### Annotation of *B. oleracea* miRNA precursors

To test our hypothesis that *B. oleracea* small reads from our three aphid gut libraries included plant miRNAs, we reannotated *B. oleracea* miRNA precursors (NCBI BioProjects: PRJNA431509, PRJNA195556, PRJNA324161, and PRJNA324144) using the recently revisited set of plant miRNA annotation criteria^18^. Prior to annotation we used FastQC v.0.11.4 to assess the quality of these datasets trimming sequences with a phred 33 score less than 28 and retaining sequences 17–25 nucleotides in length. For each of the four *B. oleracea* BioProjects, we used ShortStack v3.8.5^49^ to identify miRNA precursors against the *B. oleracea* genome^13^. In ShortStack, hairpins were limited to 300 nucleotides, and miRNA size was limited to 20–24 nucleotides^18^. We set minimum coverage at 0.25 reads per million mapped and determined the placement of reads with multiple mapping locations by considering the density of all mapped reads. ShortStack predicts miRNA precursors from a user-supplied reference genome and small RNA-seq data. The small RNAs are aligned to the genome, and areas of high coverage are identified. High coverage areas with appropriate mapping are analyzed for hairpin structures and low mismatch interactions between the mature and star miRNAs. In ShortStack, mature miRNAs are those that have the higher number of mapped reads while star miRNAs have the lower number of mapped reads. For those miRNAs annotated here, mature or star miRNA designations are assigned as such. This does not reflect the 5′/3′ naming convention.

Following annotation, we collapsed miRNA precursors from each of the four BioProjects into a single data set. Duplicates across the BioProjects were removed by retaining the duplicate with the most reads mapped to the mature miRNA. Known precursors were identified as those having e-values <10^−10^ in local BLAST searches against miRBase v22^50^. Precursors with e-values ≥10^−3^ were identified as novel. Precursors with e-values <10^−3^ and ≥10^−10^ were further scrutinized such that precursors with query coverage ≥60% were identified as known, while precursors with query coverage <60% were subject to additional BLAST searches of their mature and star sequences using local short-BLAST against miRBase v22. Short-BLAST matches with e-values <10^−10^ were identified as known, and matches with e-values ≥10^−10^ were identified as novel. Ultimate determination of novel precursors was performed following the updated plant miRNA precursor criteria found in Axtell and Myers^18^.

### miRNA target prediction

We predicted targets for *B. oleracea* miRNAs in *M. persicae* 3′ UTRs (AphidBase GFF) and in *B. oleracea* cDNA transcripts using psRNAtarget^51^. Potential miRNA targets in the aphid genome were predicted using three programs: miRanda v.3.3a^52^ (parameters: ‐sc 80 ‐go ‐8 ‐ge ‐2 ‐scale 2 ‐en 0 ‐strict ‐quiet), PITA v6^53^ (parameters: -gxp -l 7-8 -gu 7;0,8;0 -m 7;0,8;0), and RNAhybrid v2.1.2^54^ (parameters: -d <xi>, <theta> [from RNAcalibrate] -e 0 -p 0.05 -f 2,8). Targets in the plant genome were predicted using psRNAtarget^51^ and TargetFinder^55^. The two plant genome prediction programs were selected based on the analysis of Srivastava *et al*.^56^ using their optimized scoring parameters. All five miRNA target prediction programs use sequence complementarity in the seed region of the miRNA as well as the energetics of miRNA::mRNA binding to assess the ability of a miRNA to successfully bind to an mRNA. Predicted targets were manually filtered to include only those in which all respective target prediction programs overlapped in the miRNA seed region (Supplemental Fig. S1).

### Functional enrichment analysis of miRNA targets

To determine the degree of functional enrichment in the predicted miRNA targets of the aphid and plant genomes relative to the representation of genes in each genome, targets were assigned to Clusters of Orthologous Groups (COGs) using eggnog-mapper v1.0.3^57^ against EggNOG 4.5.1 orthology data^58^. 3′ UTRs targeted in the aphid genome were translated into their associated protein isoforms to be used for annotation. All isoforms sharing the 3′ UTR were included as potential targets. COG assignment was performed using Hidden Markov Models (HMMs) built from the insect (inNOG) database in EggNOG. As a comparative dataset, the entire *M. persicae* G006 proteome v2.0 (AphidBase) was run through a parallel COG annotation pipeline. In comparing these COG datasets, Monte Carlo simulations were performed to analyze the over/under-representation of target COGs relative to the reference proteome distribution. To generate null distributions, 926 3′ UTRs (the number of predicted miRNA targets, Fig. 4) were randomly sampled with replacement 10^6^ times from the reference proteome. The 3′ UTRs were then expanded into their associated isoforms, and the number of proteins was added to the distribution allowing more than 926 proteins to be included in a distribution for the aphid proteome. A comparison was then made between each null COG distribution and the associated miRNA target COG value. Two-tailed p-values were calculated empirically from the null distributions to record significance in the event that the miRNA target COG category was found to be as or more extreme than the null distribution such that p < 0.05.

For the miRNA targets in the plant genome, the same functional enrichment analysis was repeated. Plant cDNA sequences were translated within EggNOG, and COG assignment was performed using HMMs built from the viridiplantae (virNOG) database in EggNOG. As a comparative dataset, the entire *B. oleracea* cDNA library (psRNAtarget^51^) was run through a parallel COG annotation pipeline. To produce Monte Carlo simulations that complement the aphid proteome analysis, 576 cDNA transcripts (the number of predicted miRNA targets, Fig. 4) were randomly sampled with replacement 10^6^ times. Comparisons and p-values were calculated as described above.

### Assessment of *B. oleracea* miRNA precursor processing

The secondary structure of each precursor was produced using RNAfold v2.4.4^59^ and visualized using VARNA v3-93^60^ (Supplementary Document S2). These computational predictions validated the mature miRNA, star miRNA, loop, 5′ end and 3′ end regions of each miRNA precursor as previously determined through ShortStack. Next, we used Bowtie2 v2.2.6 to align our *M. persicae* gut small RNA data against miRNA precursors of the 32 *B. oleracea* miRNAs present in our gut datasets, filtering alignments to exclude any with mismatches or gaps (Supplementary Document S1). Following alignment and allowing for up to one nucleotide overlap with an adjacent region, reads were assigned to one of the following five regions: mature miRNA, star miRNA, loop, 5′ end or 3′ end. Any read that failed to meet these criteria was considered to be a “precursor fragment”.

### Ethical approval and informed consent

Not required. The work did not involve vertebrates.

## Supplementary information

Supplementary information

Supplementary information

Supplementary information

Supplementary information

Supplementary information

Supplementary information

Supplementary information

## Data Availability

All data sets supporting the conclusions are published in the article and the online supporting information. The small RNA next‐generation sequencing data sets are available at NCBI Sequence Read Archive (SRA) under BioProject PRJNA395356. The Python scripts written to perform analysis and figure production are also available on the WilsonLabMiami GitHub Repository: Thompson_etal_miRNAaphidgut (https://github.com/WilsonLabMiami/Thompson_etal_miRNAaphidgut).
