# Supplementary material for: The green peach aphid gut contains host plant microRNAs identified by comprehensive annotation of Brassica oleracea small RNA data
Source: Sci Rep. 2019 Dec 11;9:18904. doi: 10.1038/s41598-019-54488-1 (PMC6906386; doi:10.1038/s41598-019-54488-1)
Supplement: Supplementary file 1 — Supplementary information [file 41598_2019_54488_MOESM1_ESM.pdf]

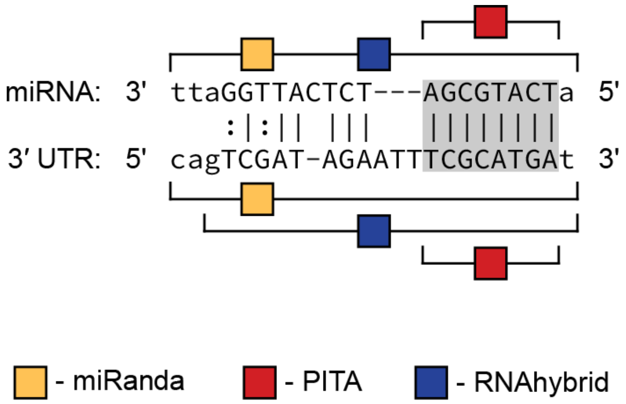

Supplemental Figure 1. miRNA Target site selection methodology
Intersect of three miRNA target prediction programs wherein the plant miRNA (bol-miR_novel_70) is shown to be able to target this site on an aphid mRNA 3′-UTR (LOC111034866).

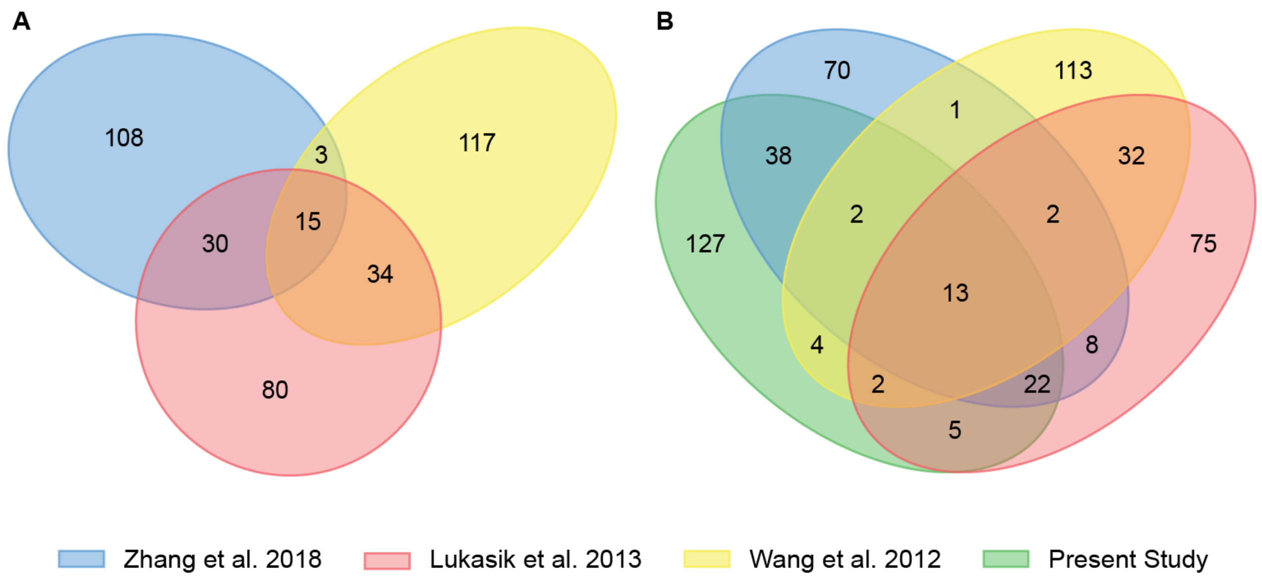

Supplemental Figure 2. Agreement between previous *Brassica oleracea* miRNA annotations

(A) Proportional Venn diagram showing the agreement between published miRNA sequences of three recent *B. oleracea* miRNA annotations. (B) Venn diagram of agreement between sets in A as well as this study.
