# Supplementary material for: The green peach aphid gut contains host plant microRNAs identified by comprehensive annotation of Brassica oleracea small RNA data
Source: Sci Rep. 2019 Dec 11;9:18904. doi: 10.1038/s41598-019-54488-1 (PMC6906386; doi:10.1038/s41598-019-54488-1)

**The green peach aphid gut contains host plant microRNAs identified by comprehensive annotation of *Brassica oleracea* small RNA data**

Max C. Thompson, Honglin Feng, Stefan Wuchty, and Alex C. C. Wilson

Representation of *M. persicae* gut small RNA reads that map to *B. oleracea* miRNA precursors with miRNAs found in aphid gut. Reads mapped are exact matches. Red reads are perfect star miRNA matches. Green reads are perfect mature miRNA matches. X-axis indicates precursor nucleotide position, miRNA name and precursor identity in parentheses.


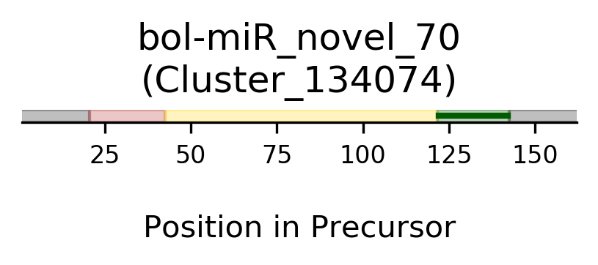

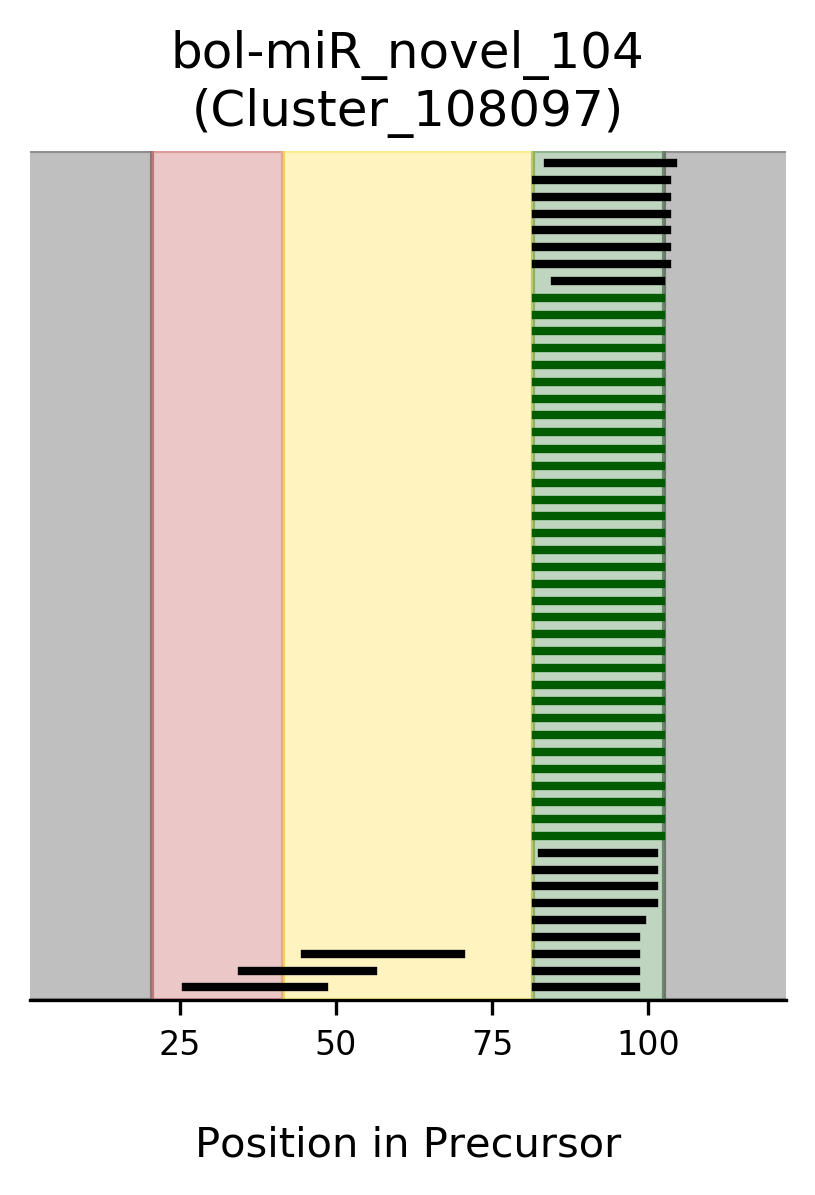

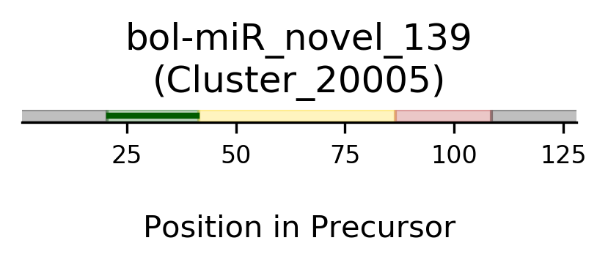

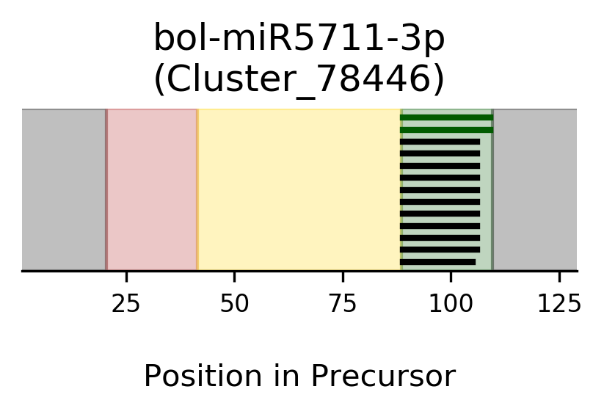

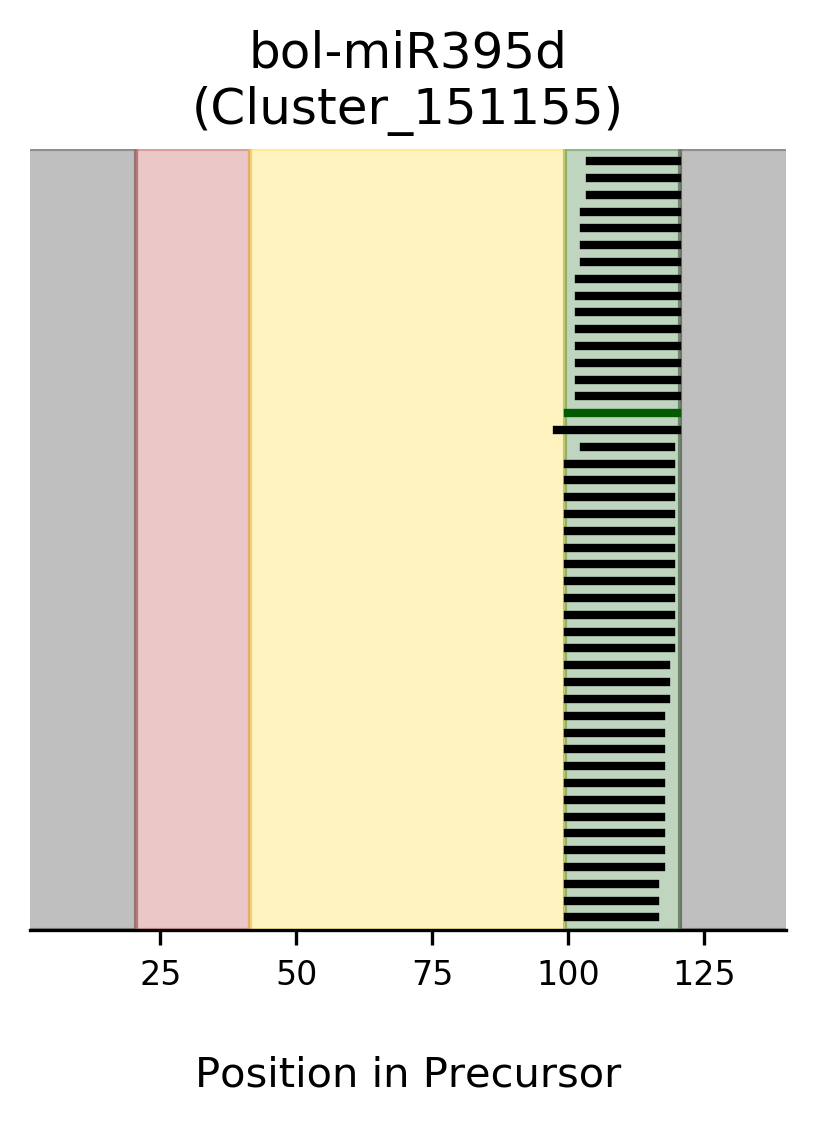

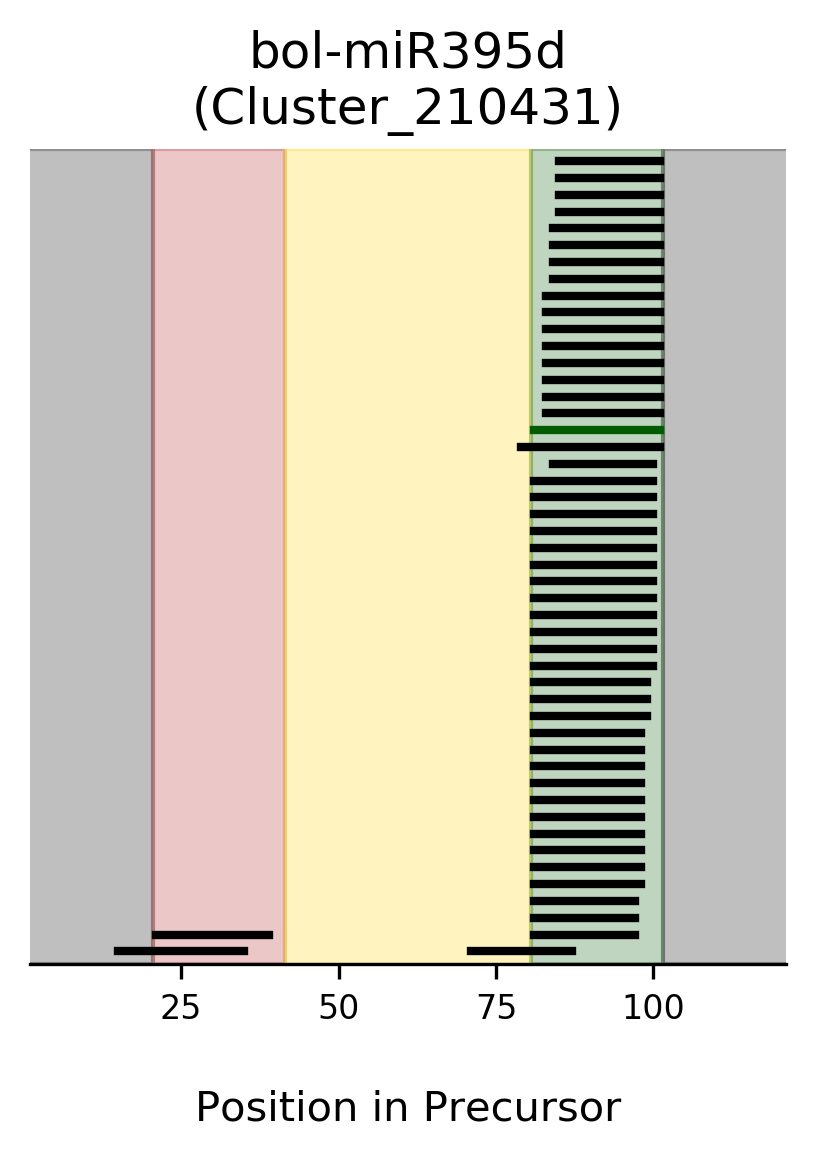

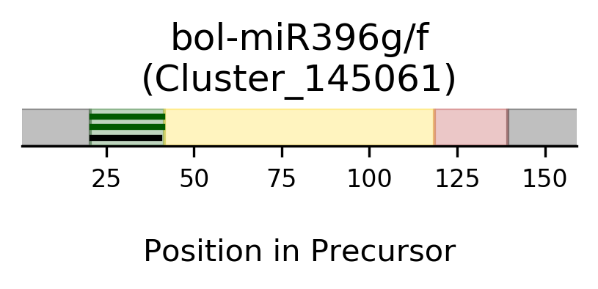

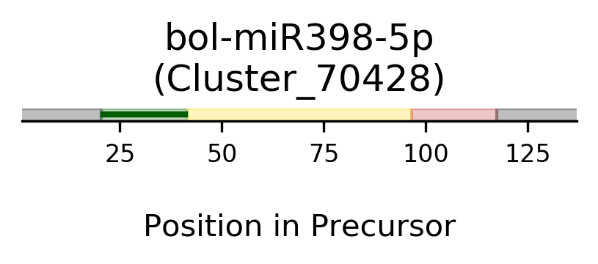

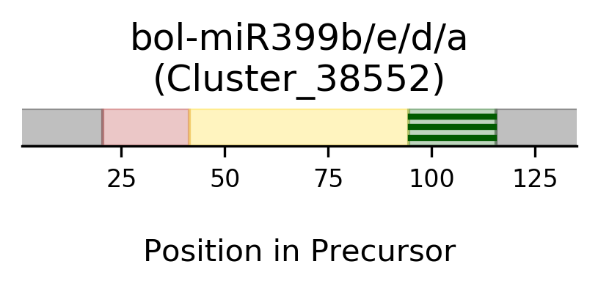

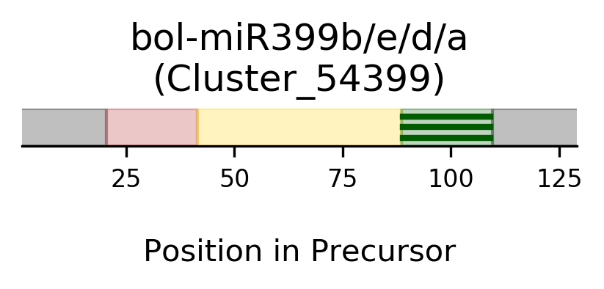

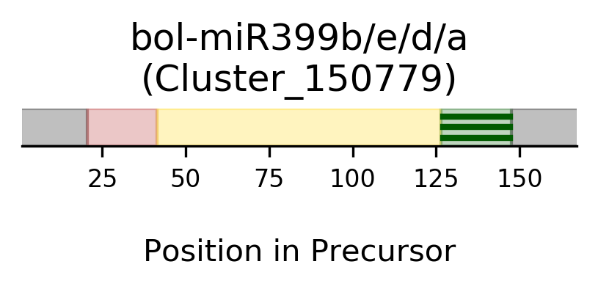

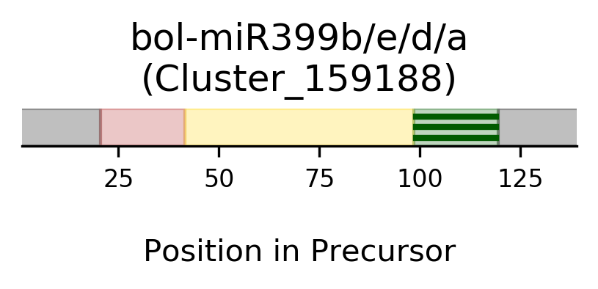

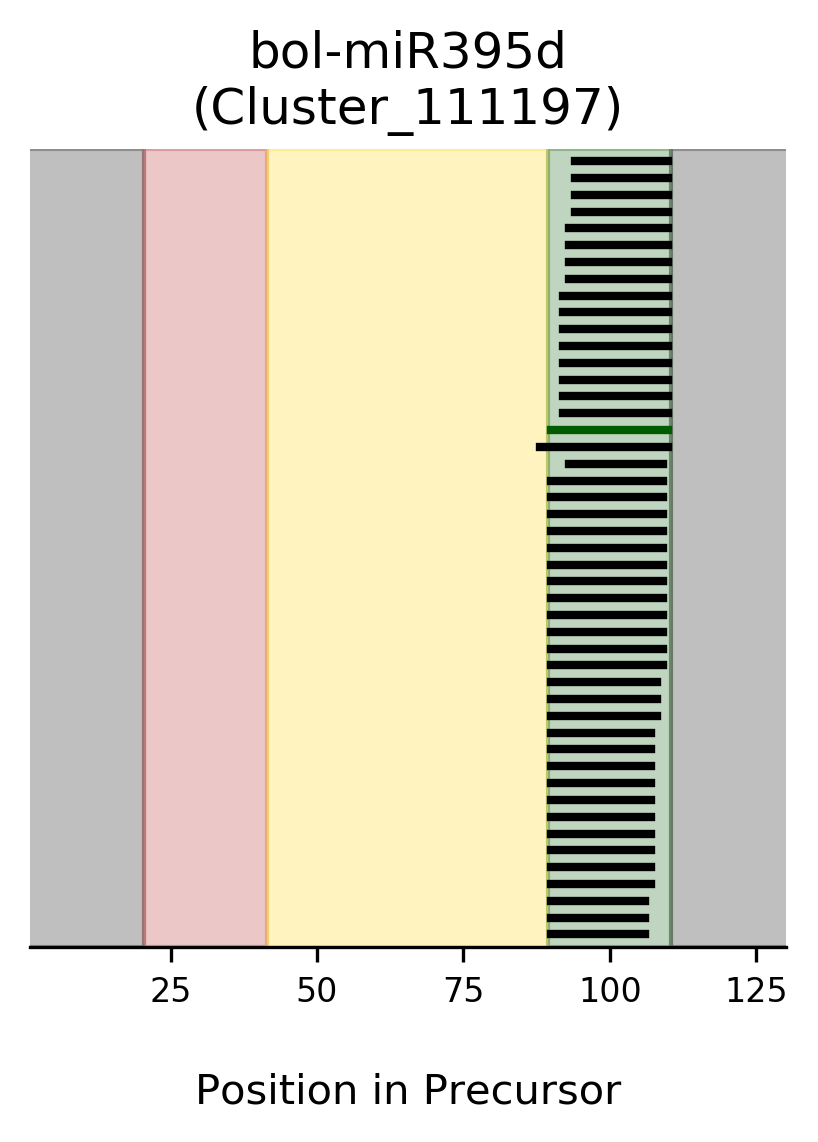

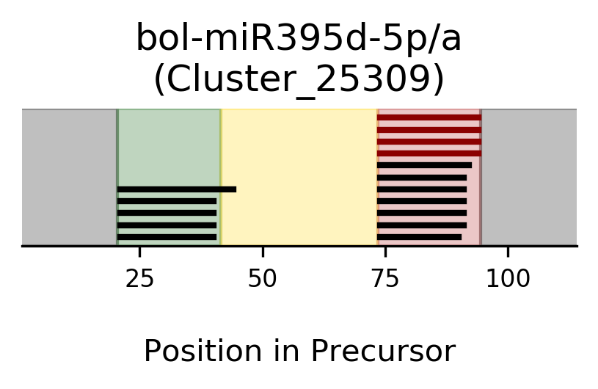

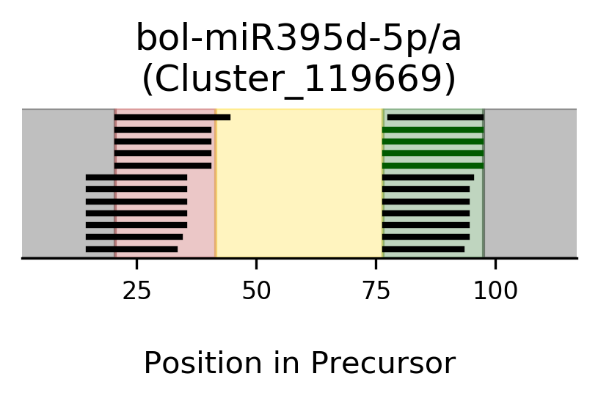

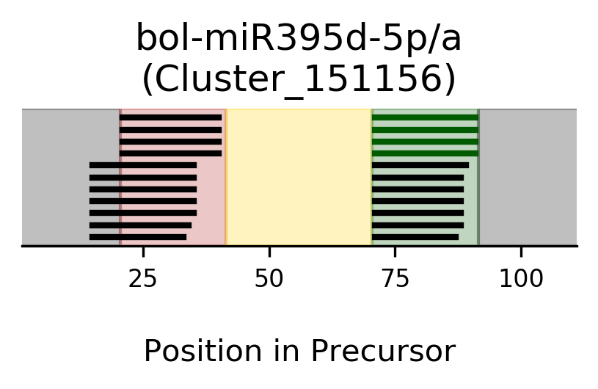

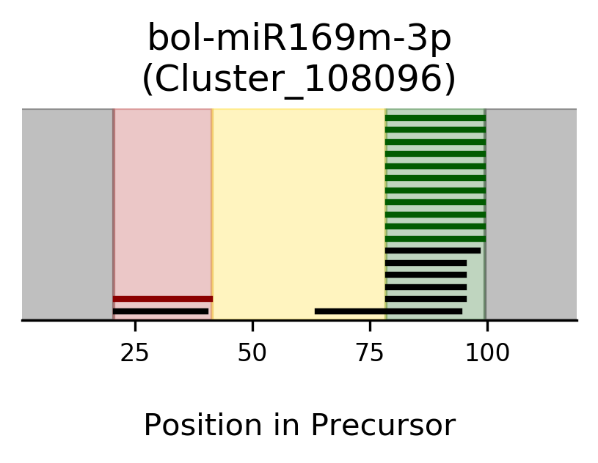

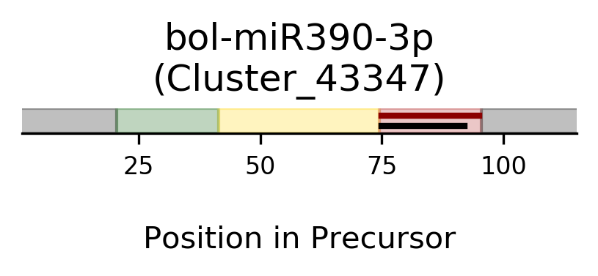

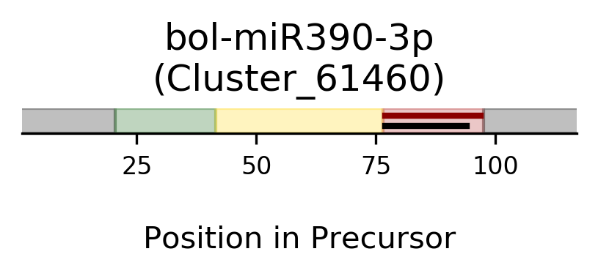

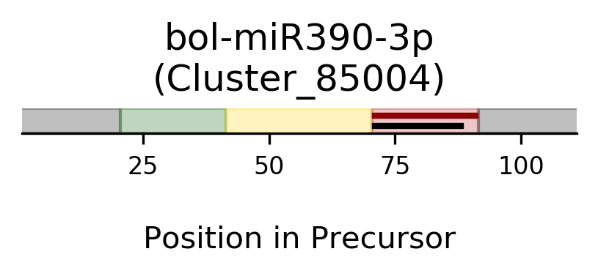

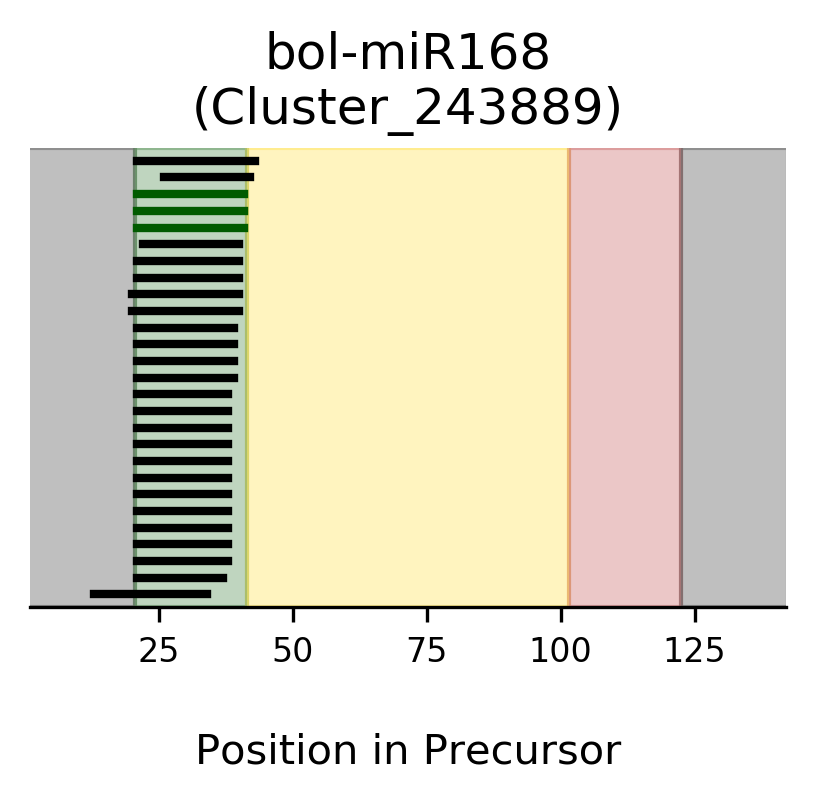

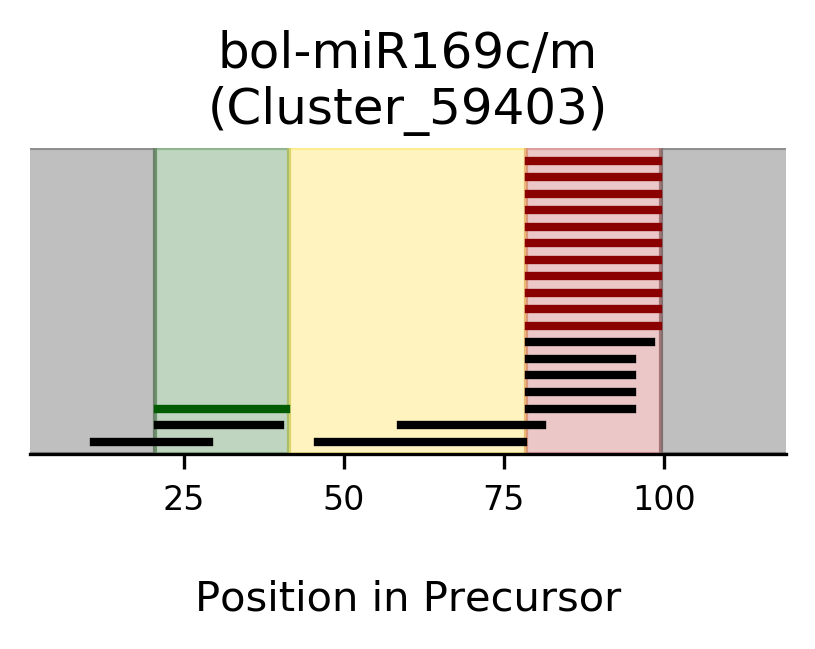

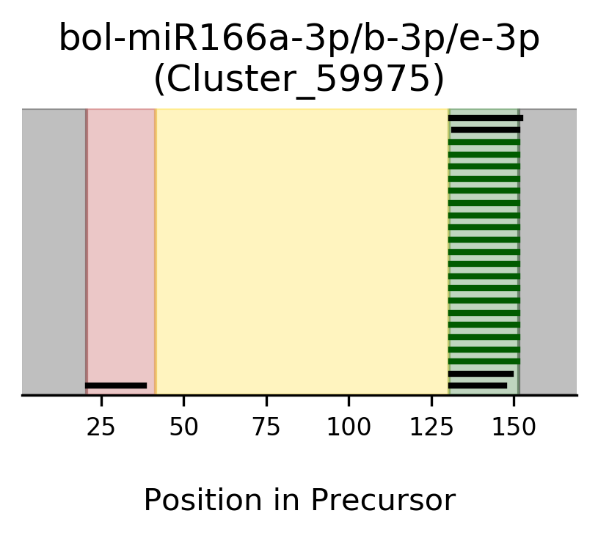

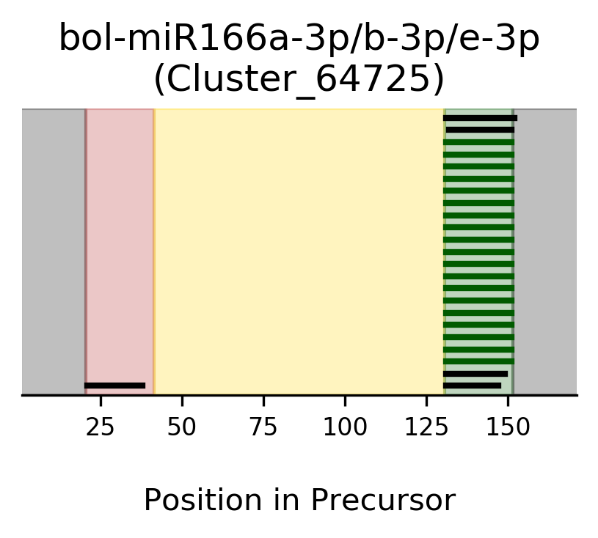

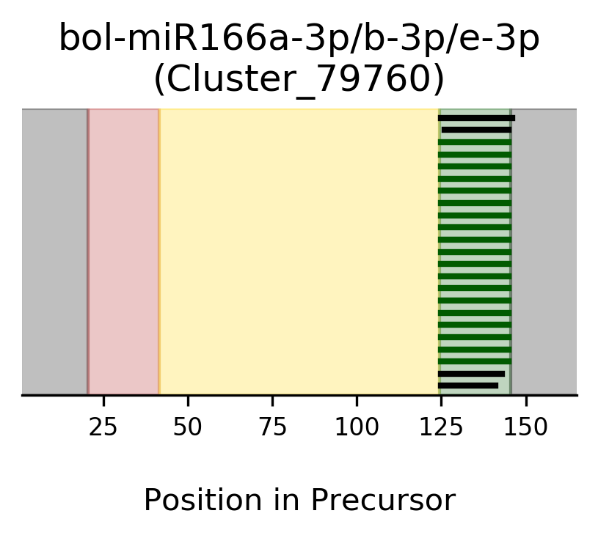

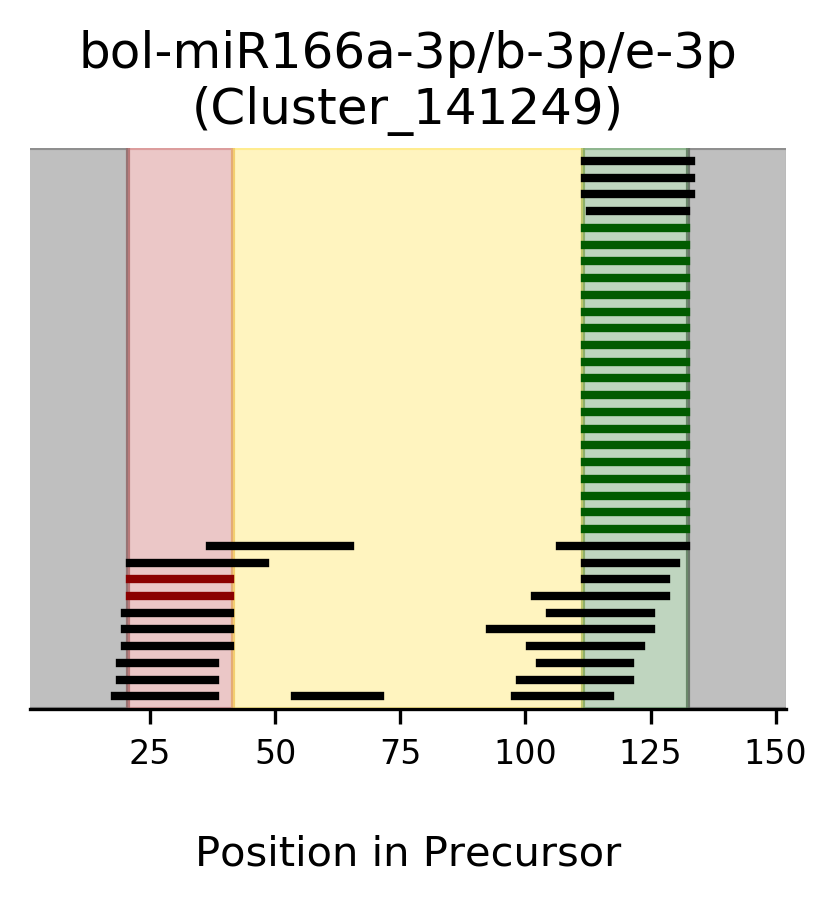

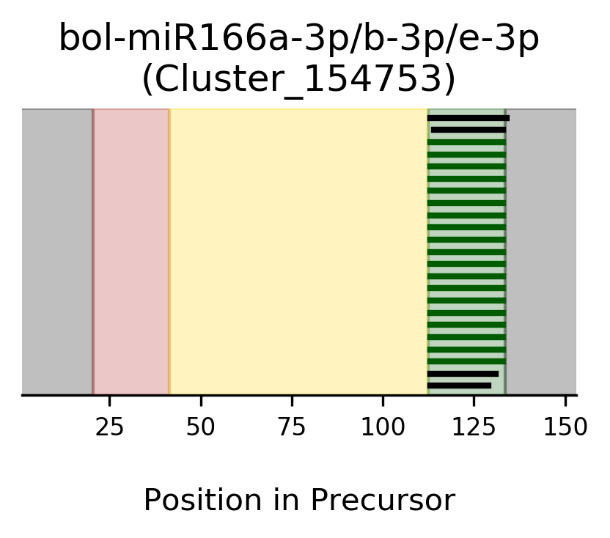

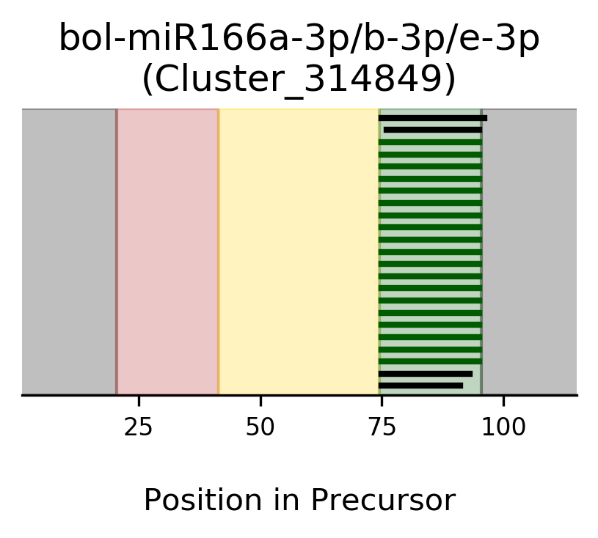

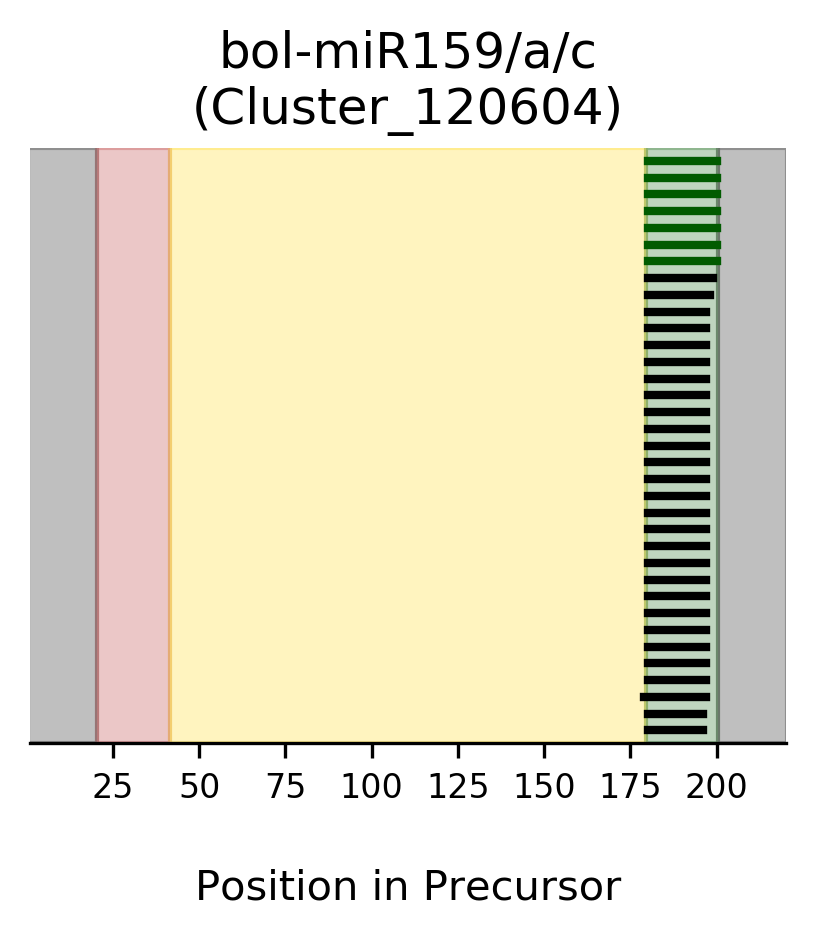

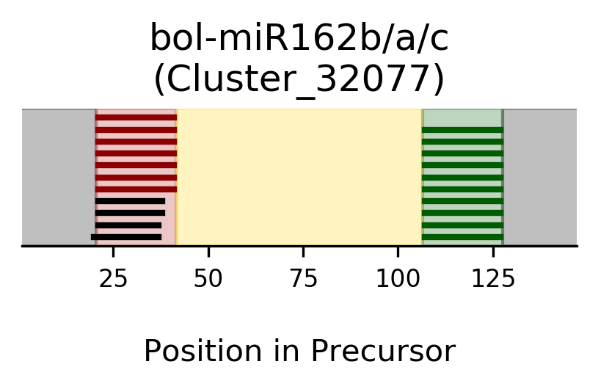

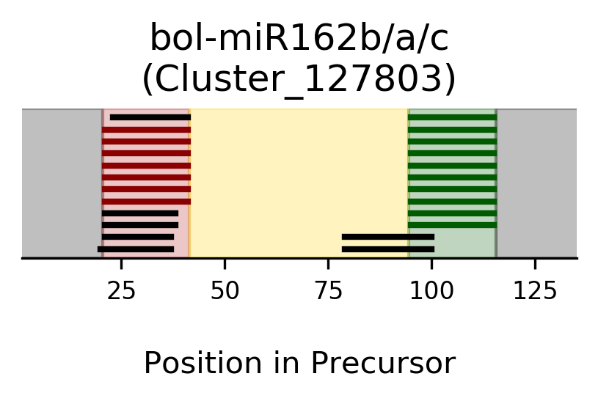

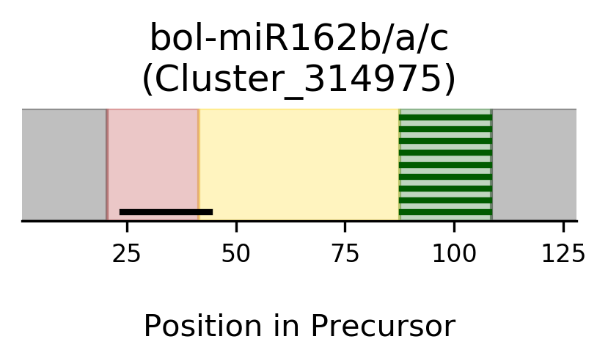

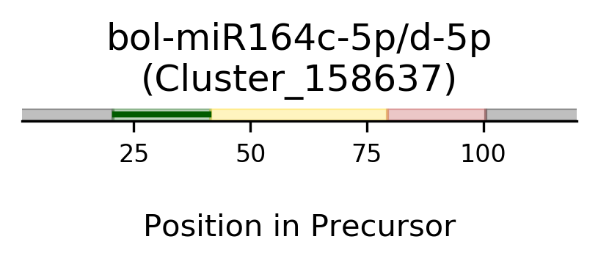

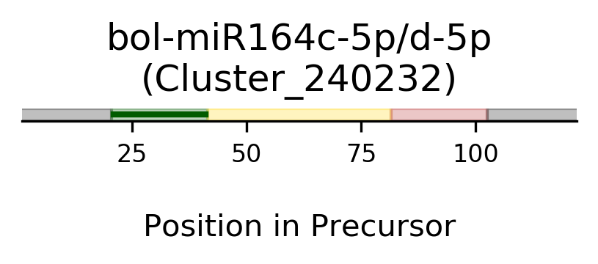

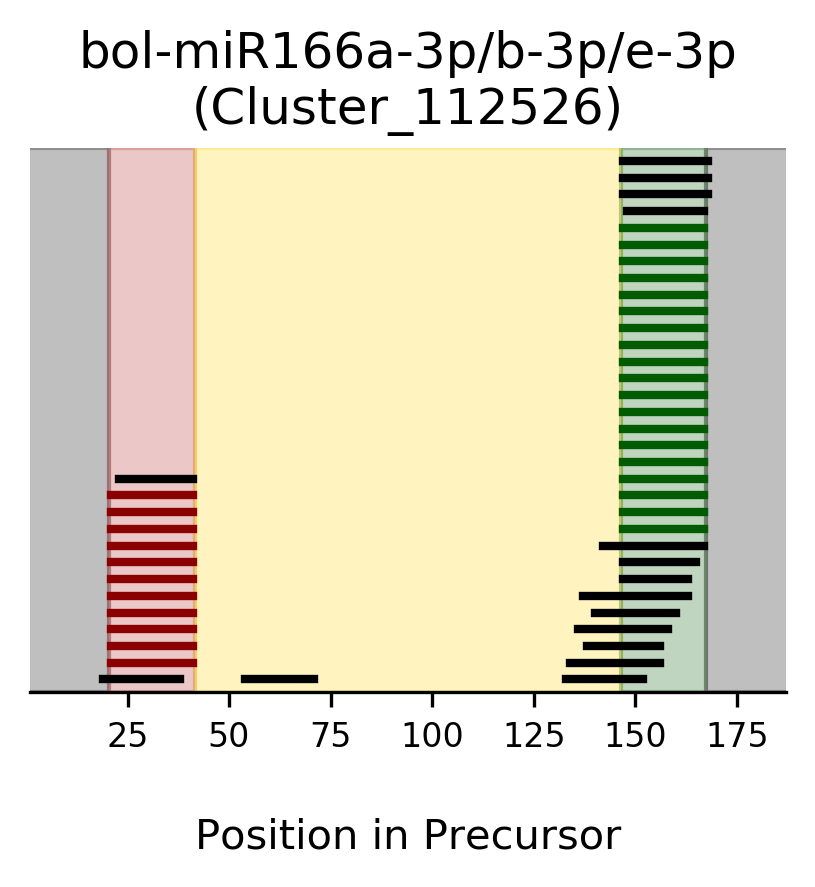

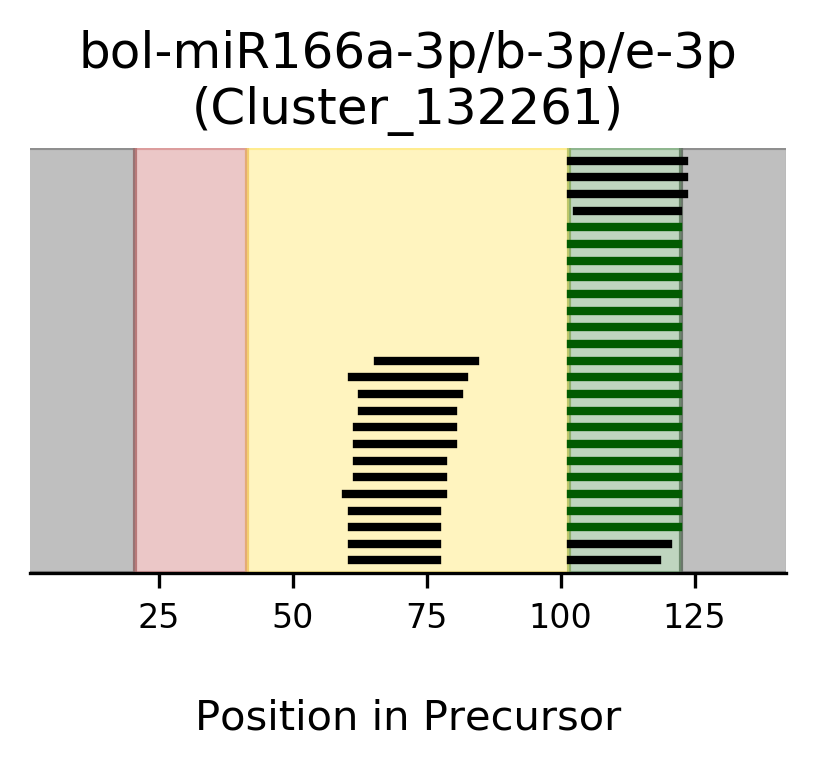

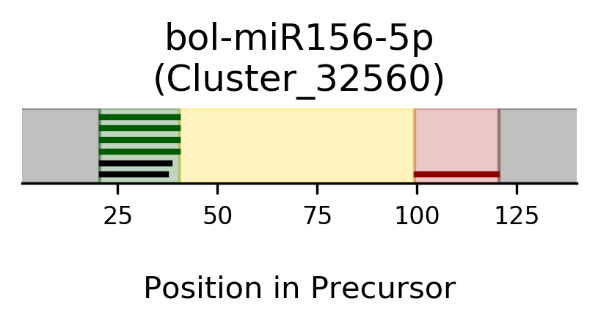

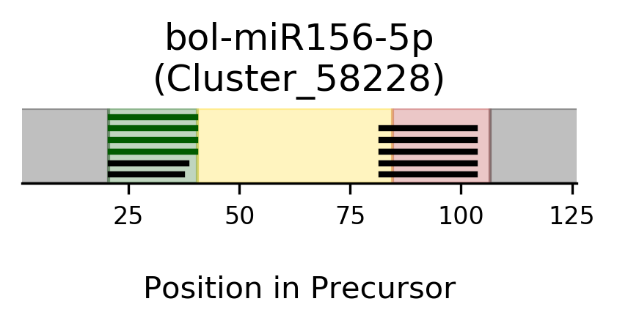

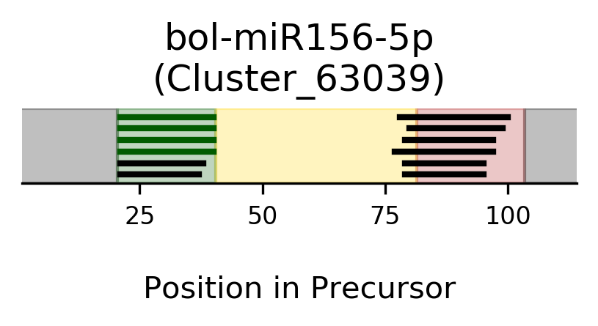

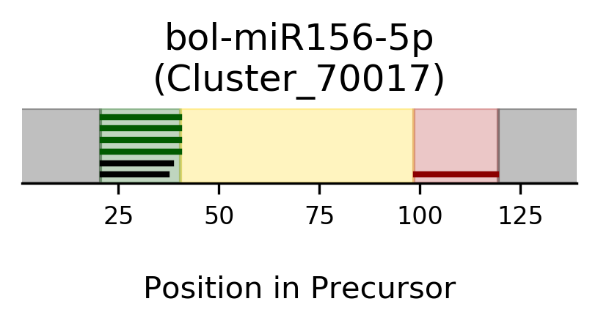

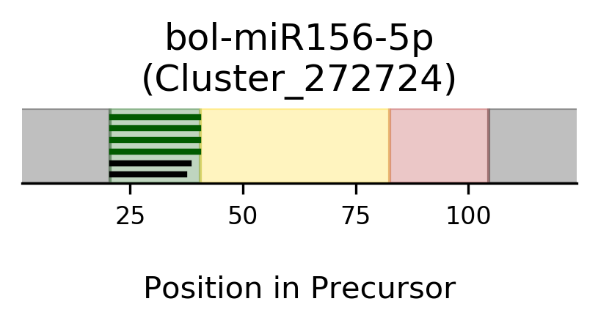

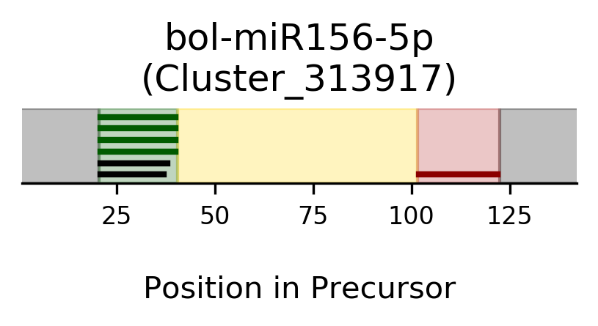

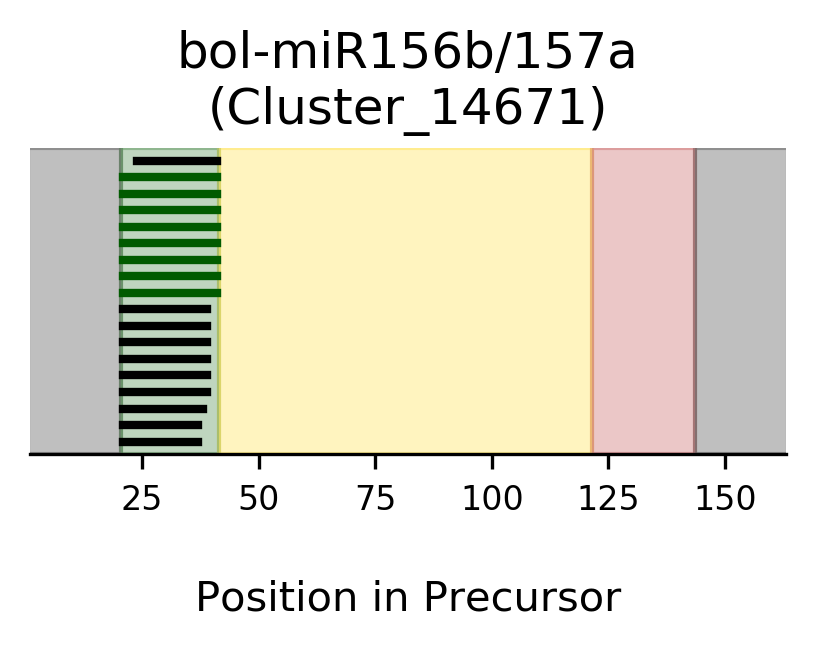

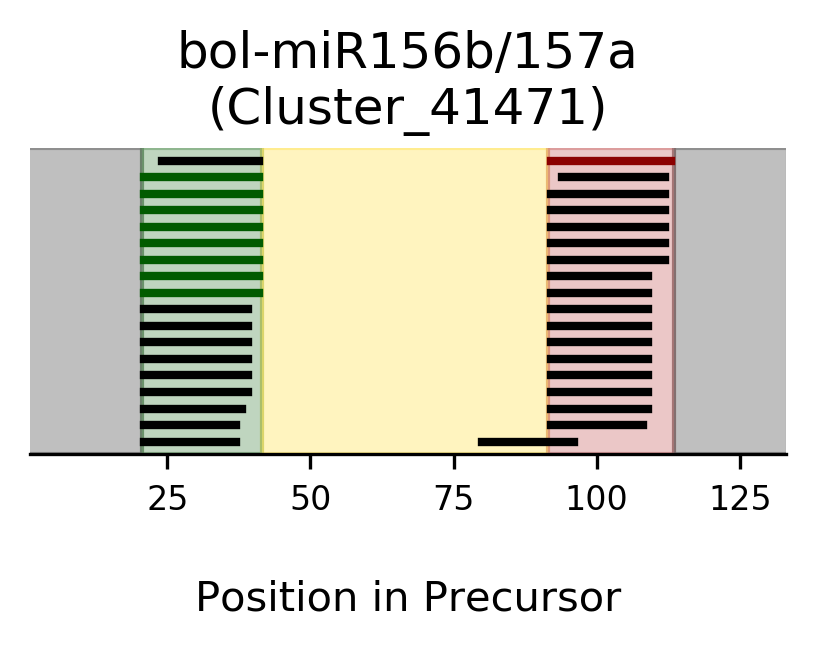

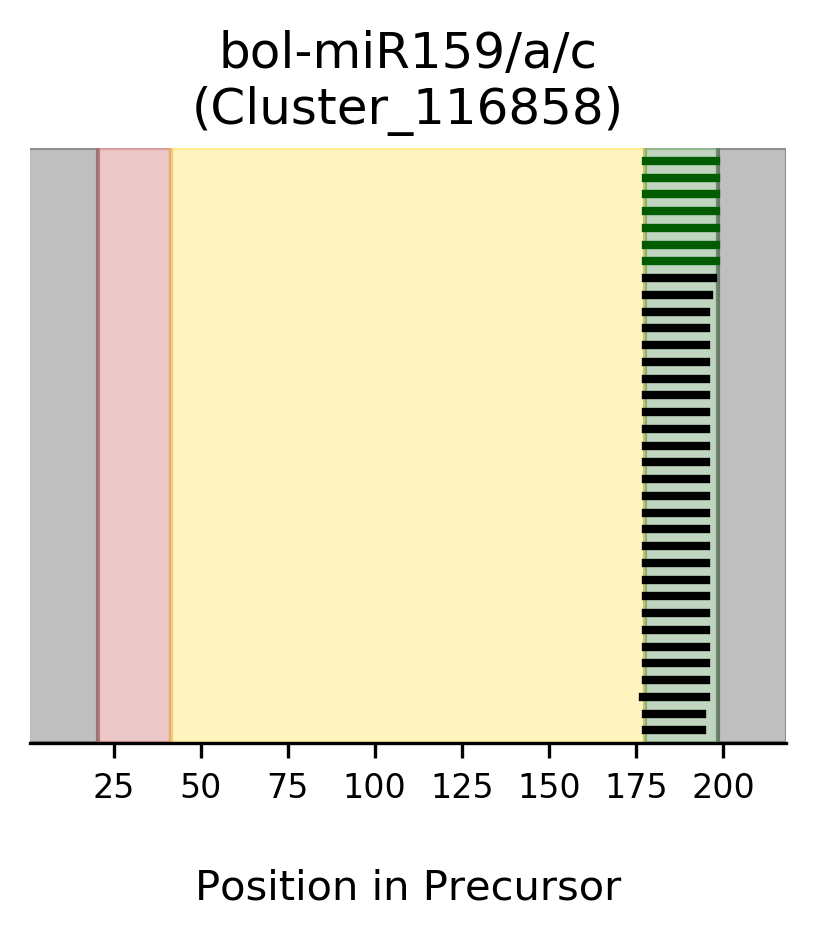

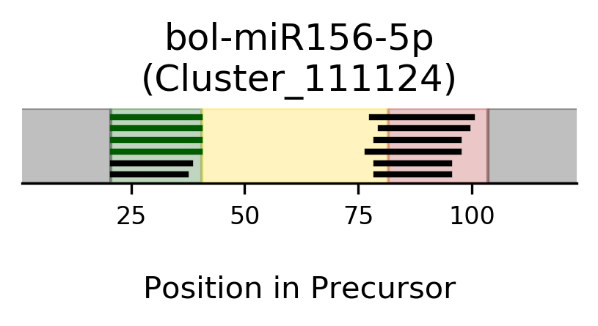

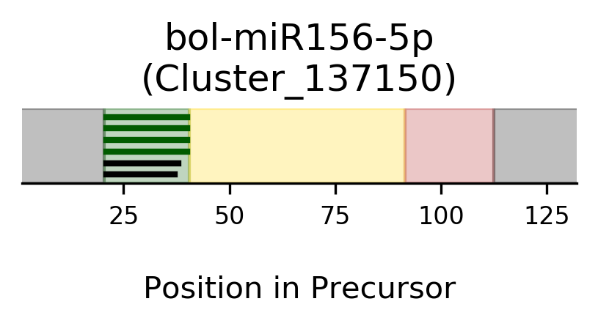

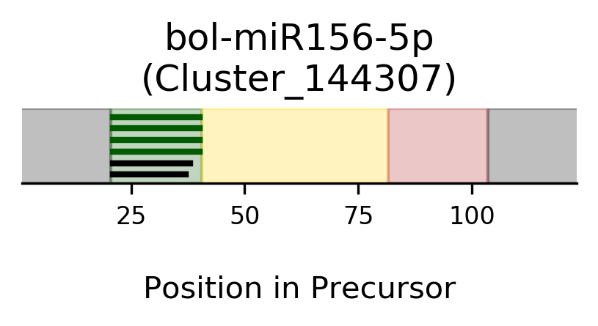


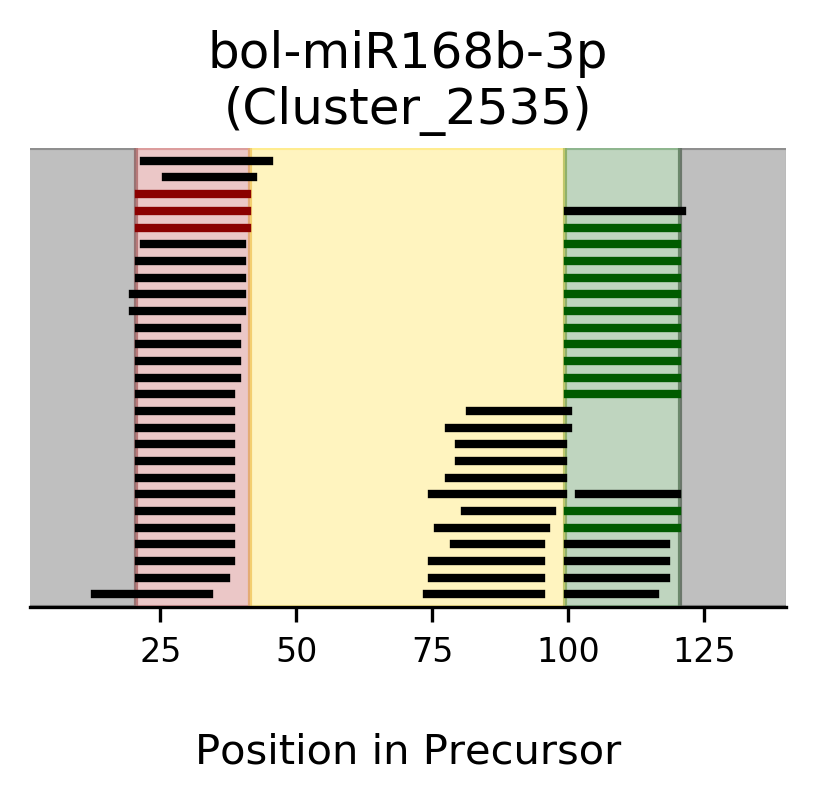

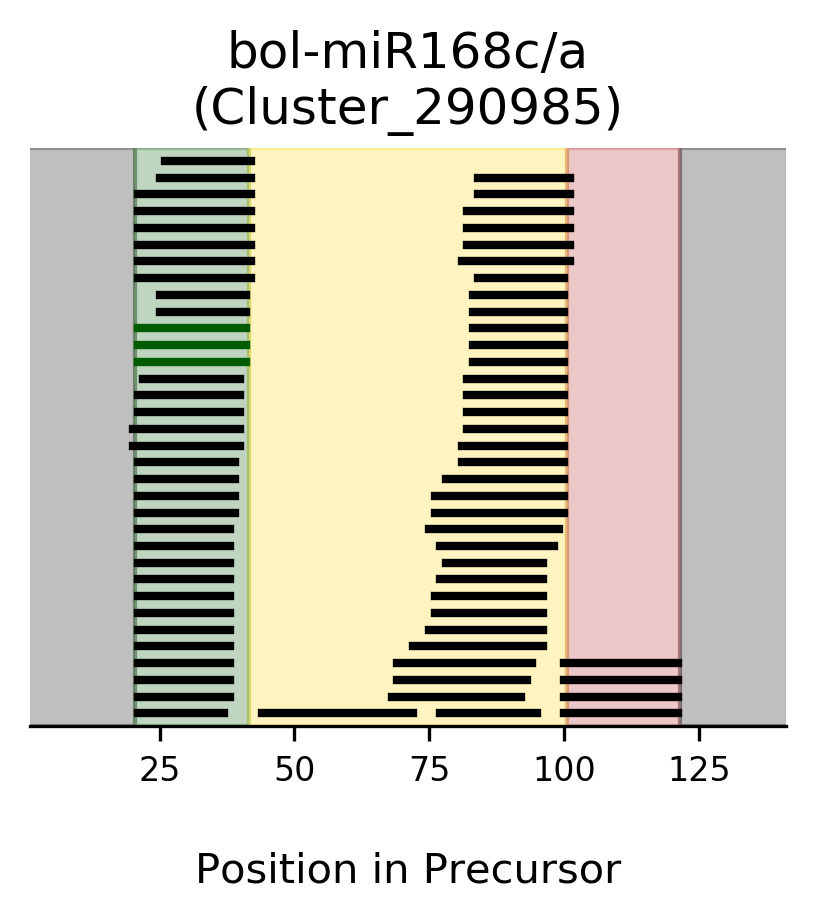


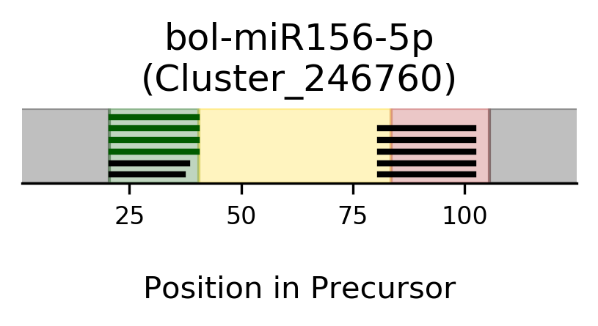

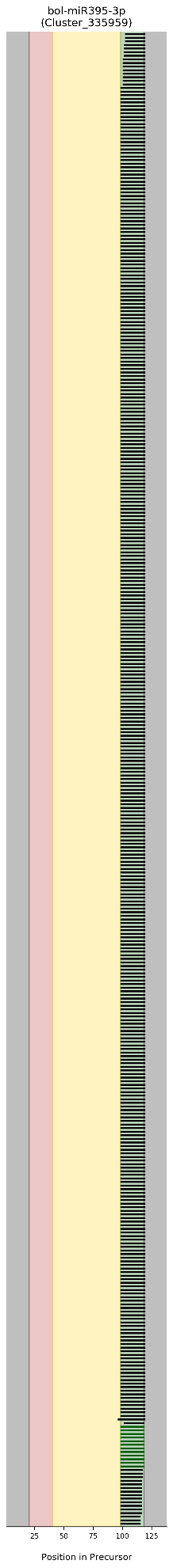

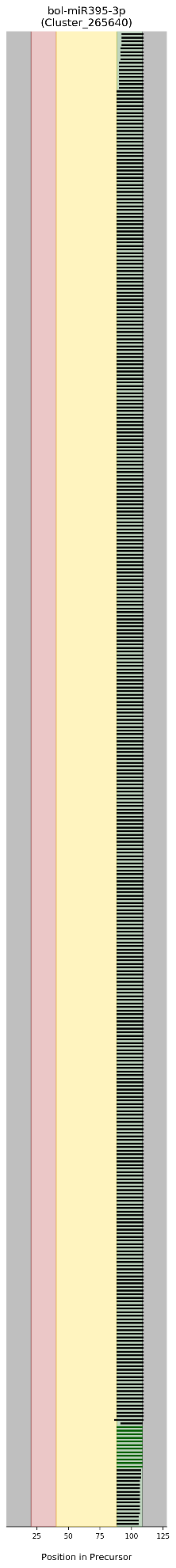

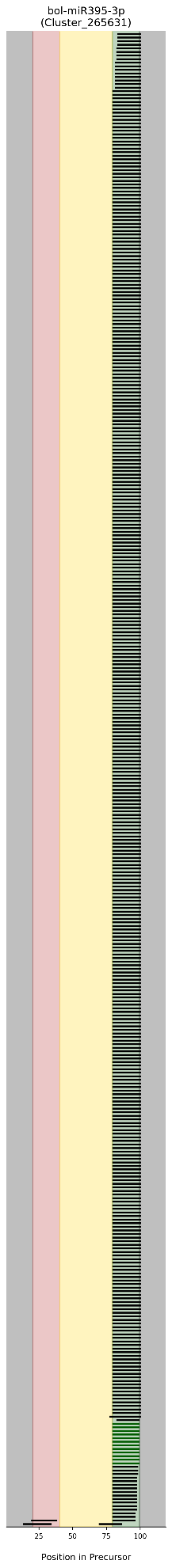

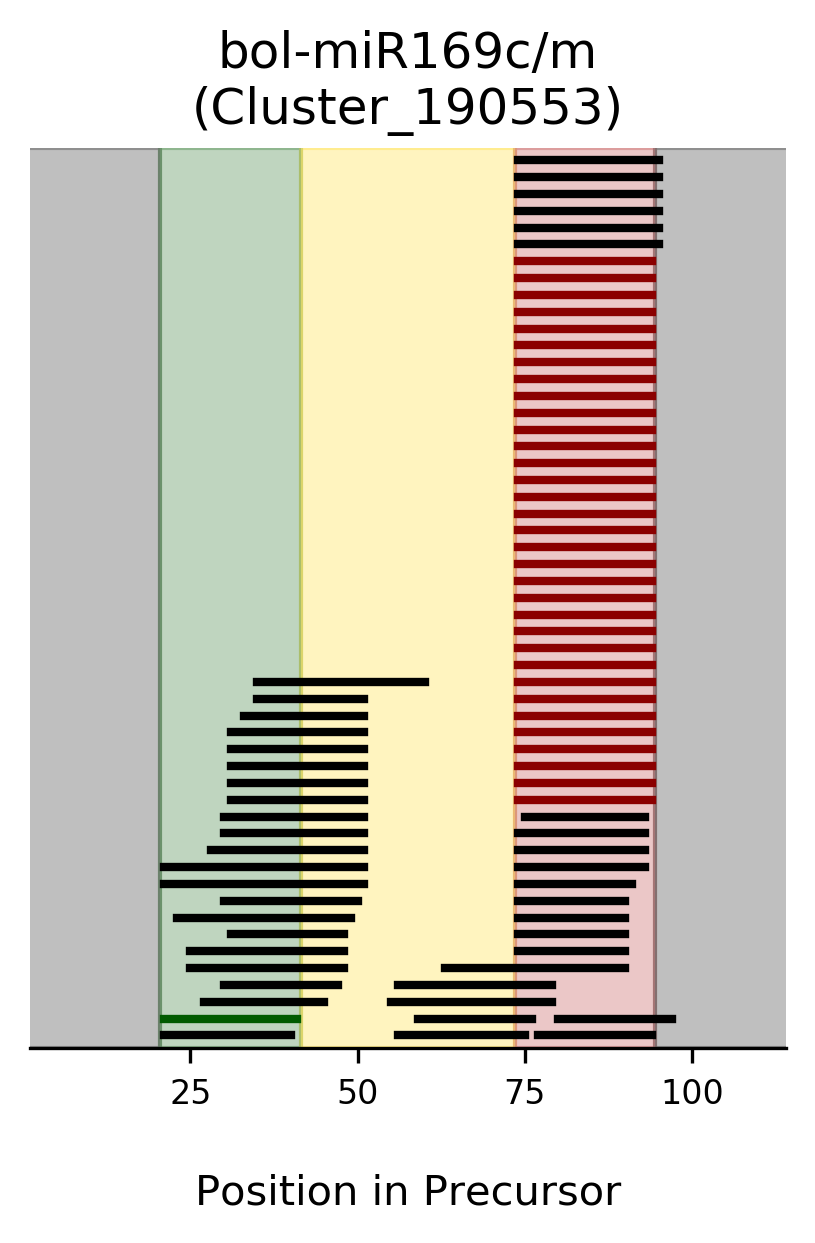

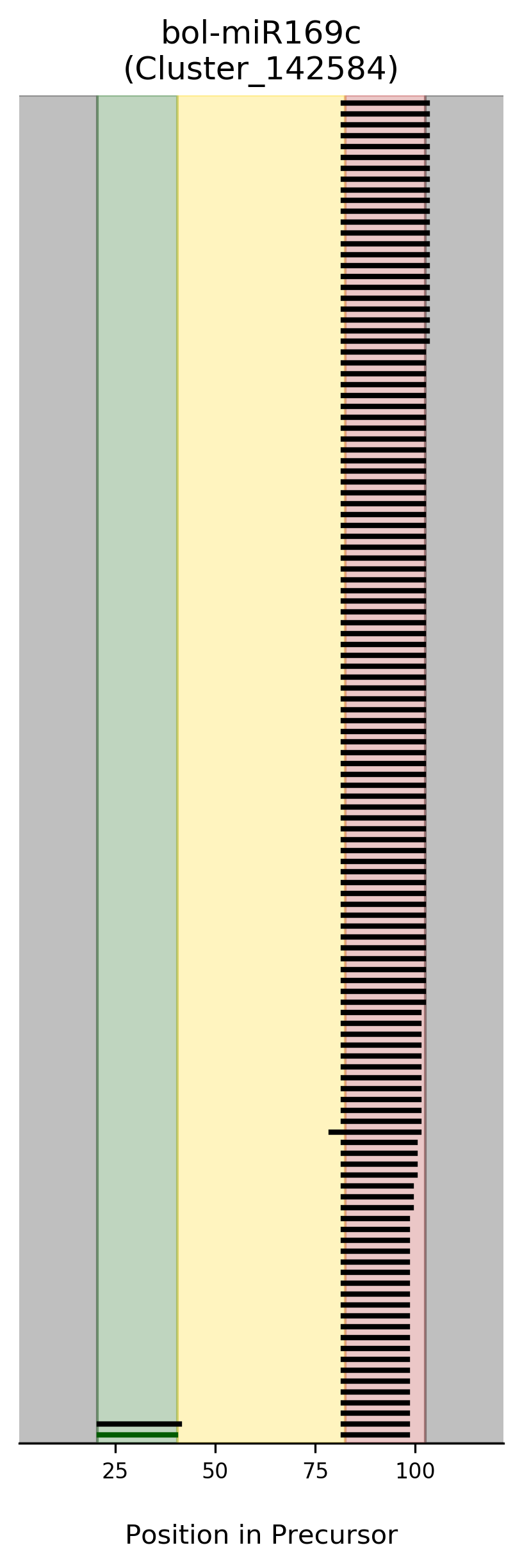

Supplement: Supplementary file 6 — Supplementary information [file 41598_2019_54488_MOESM6_ESM.docx]
