# Supplementary material for: The green peach aphid gut contains host plant microRNAs identified by comprehensive annotation of Brassica oleracea small RNA data
Source: Sci Rep. 2019 Dec 11;9:18904. doi: 10.1038/s41598-019-54488-1 (PMC6906386; doi:10.1038/s41598-019-54488-1)
Supplement: Supplementary file 7 — Supplementary information [file 41598_2019_54488_MOESM7_ESM.docx]

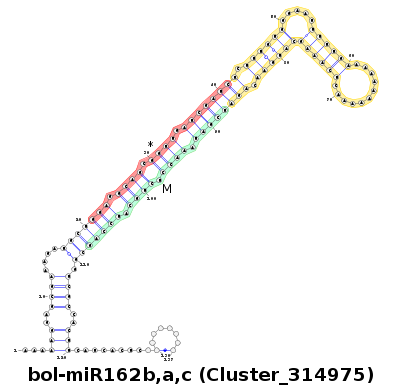

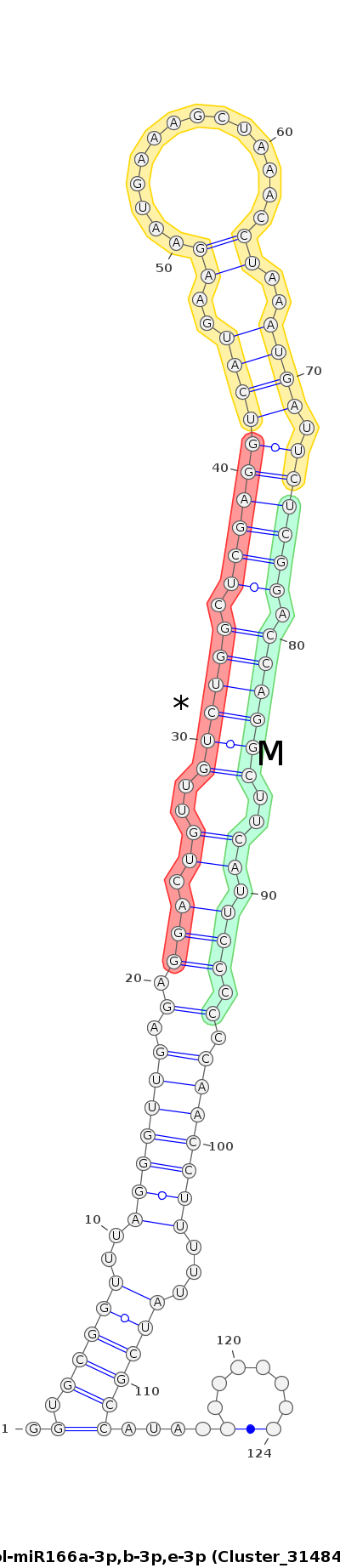

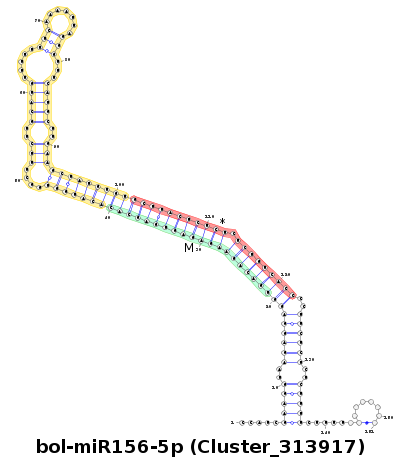

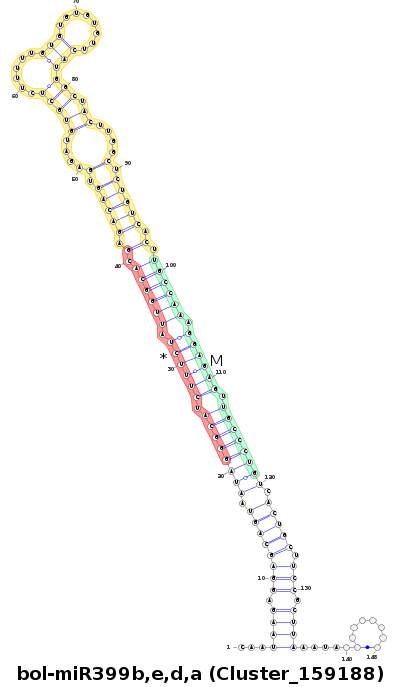

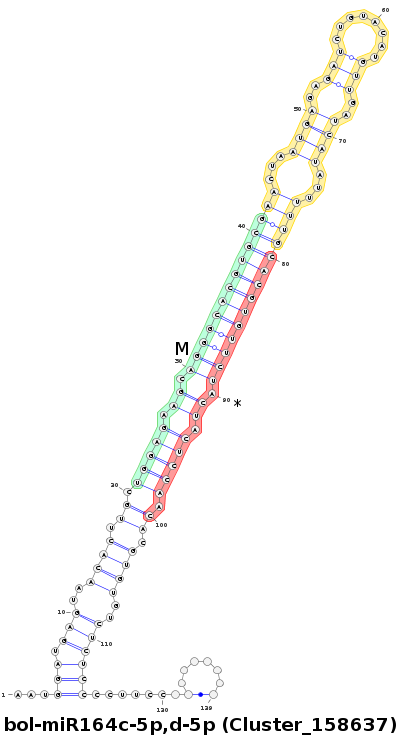

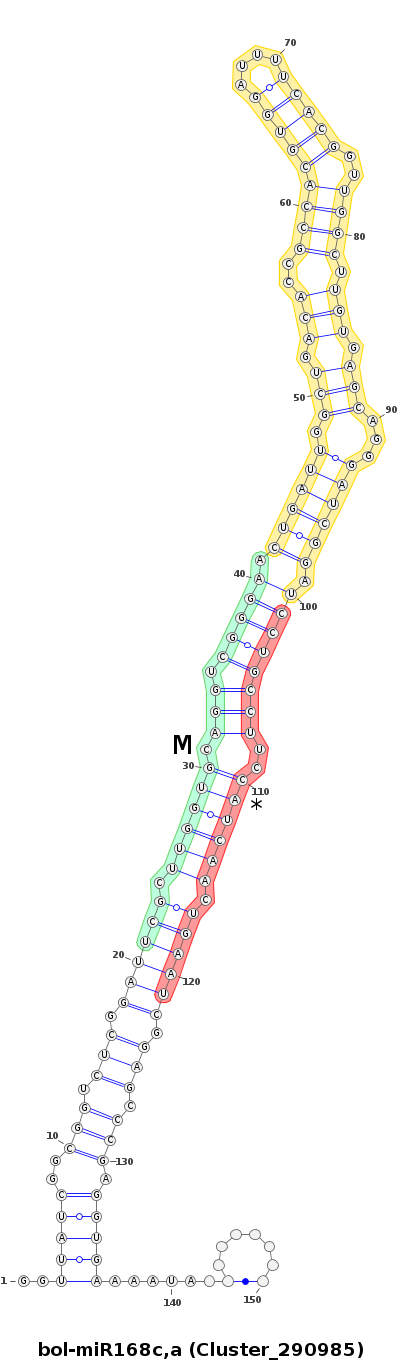

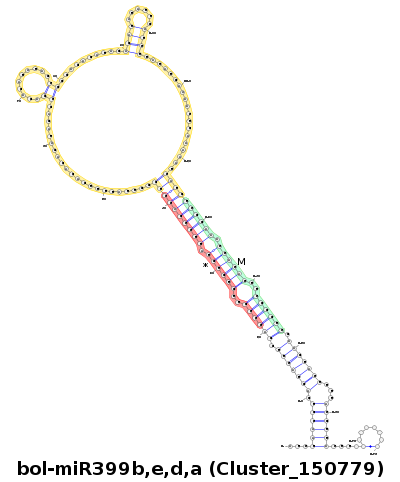

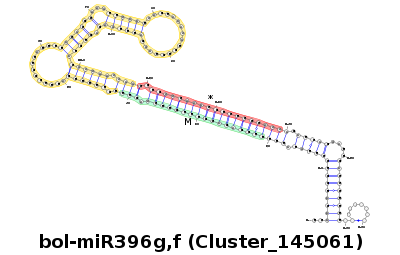

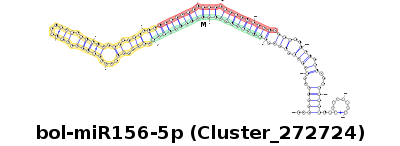

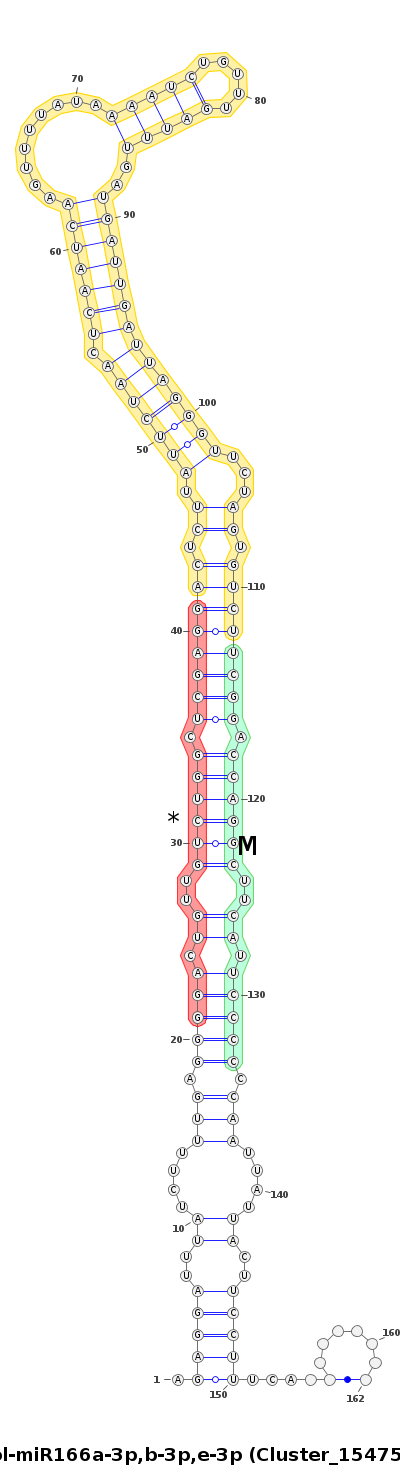

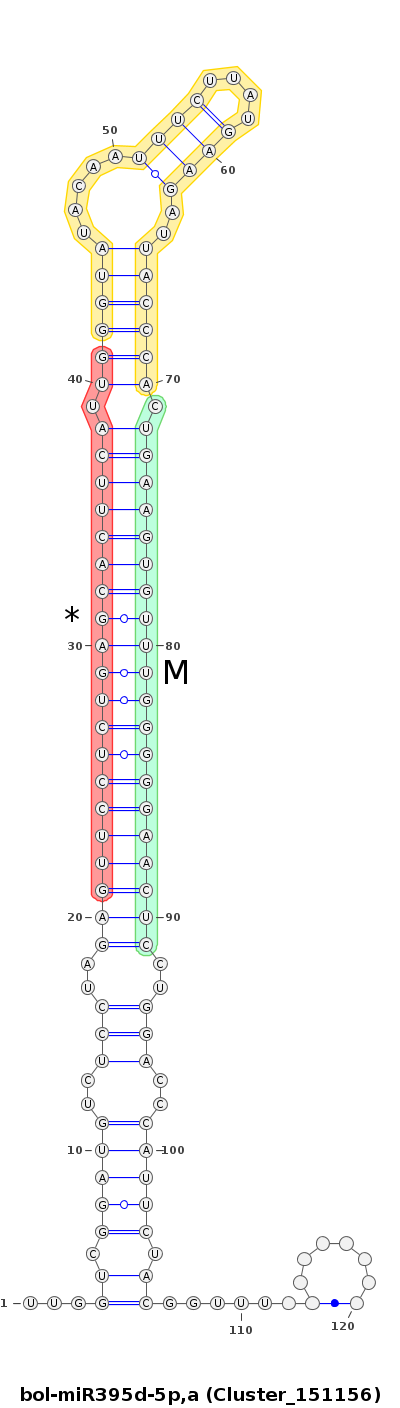

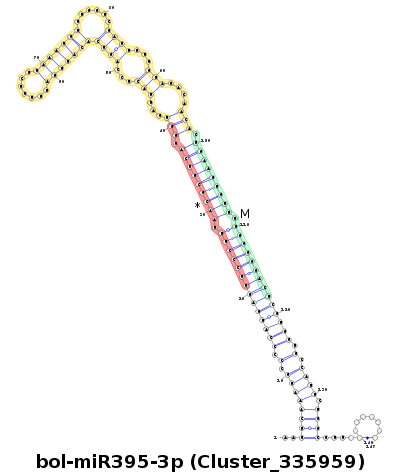

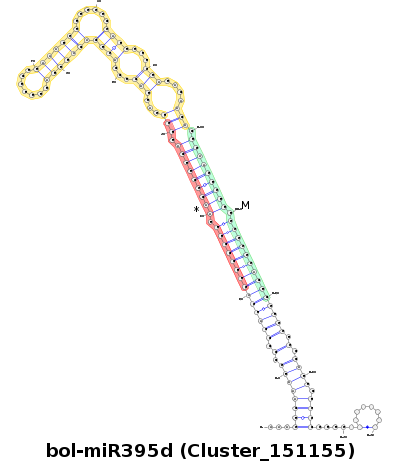

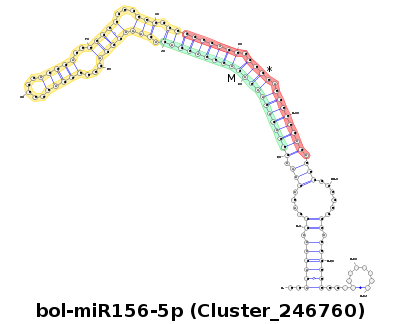

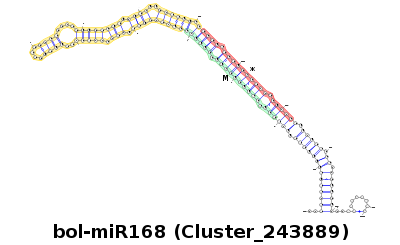

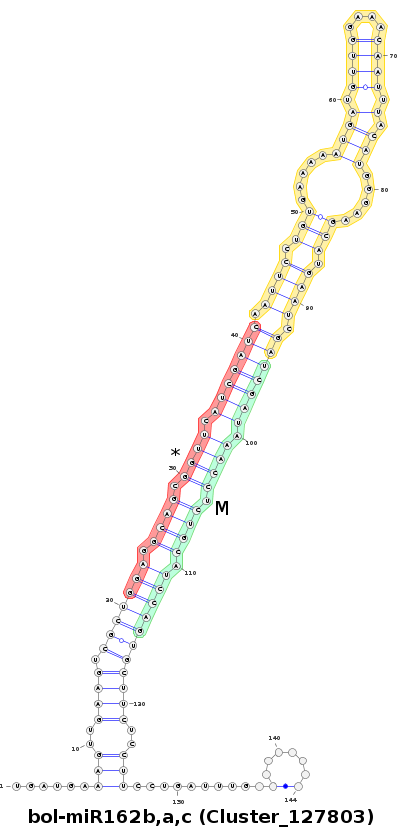

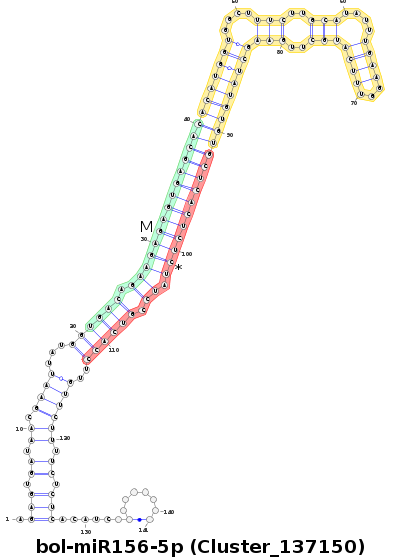

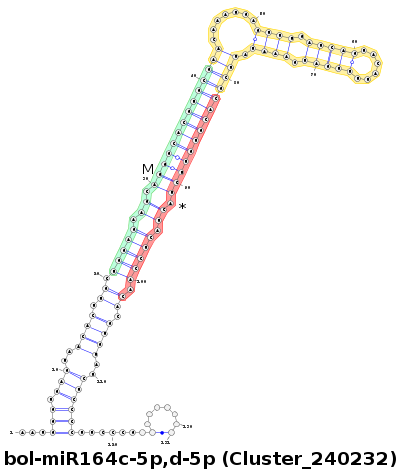

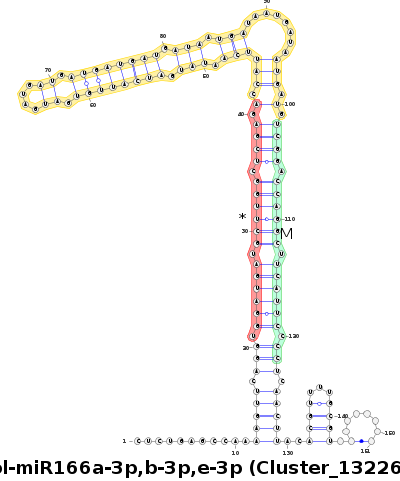

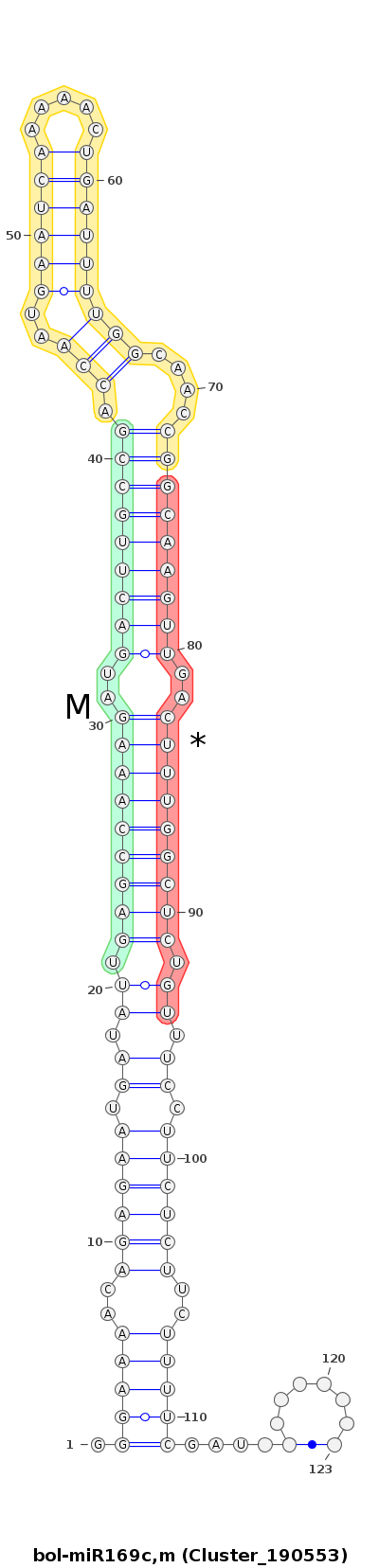

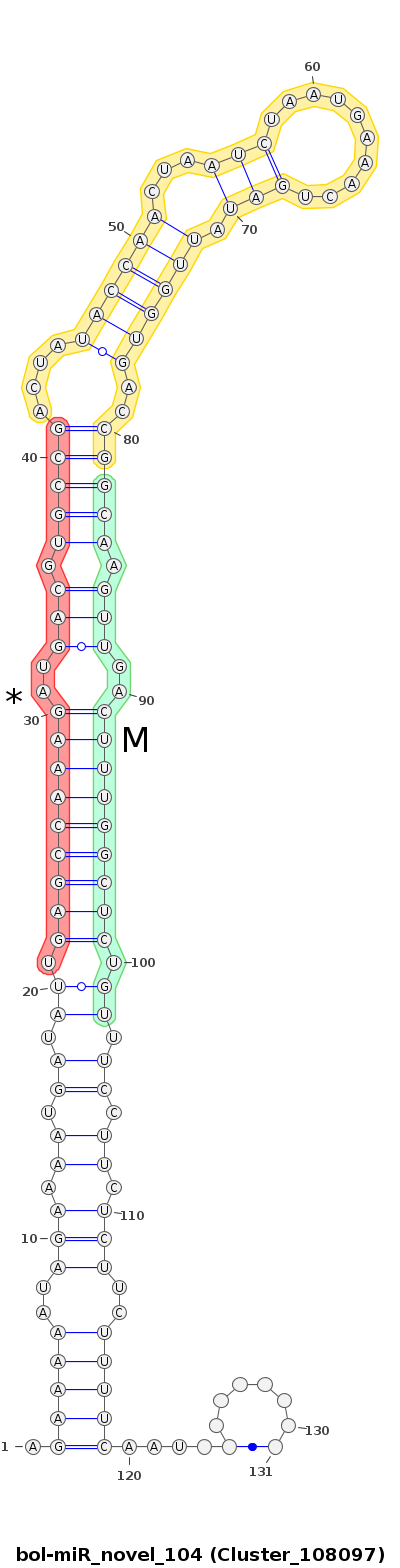

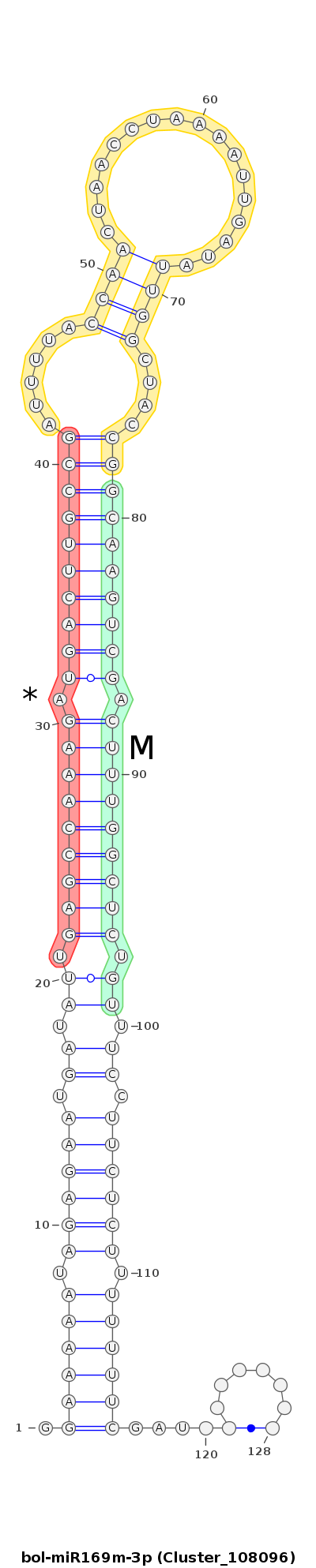

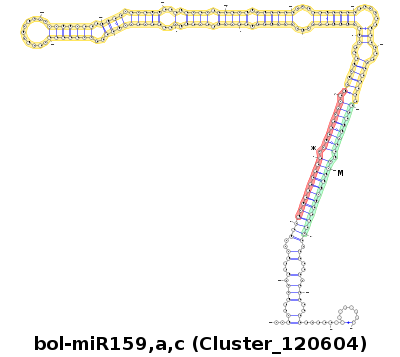

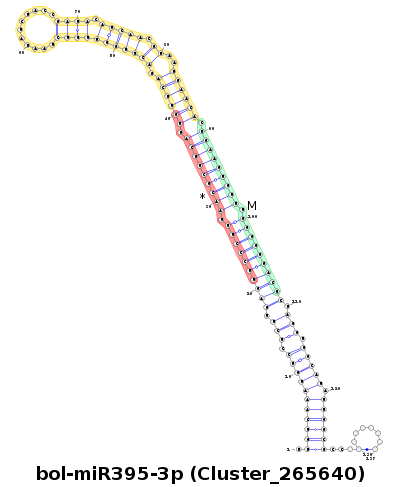

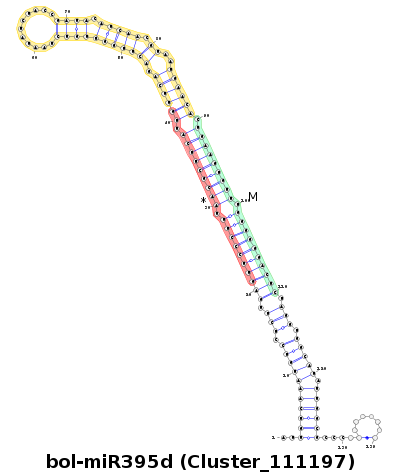

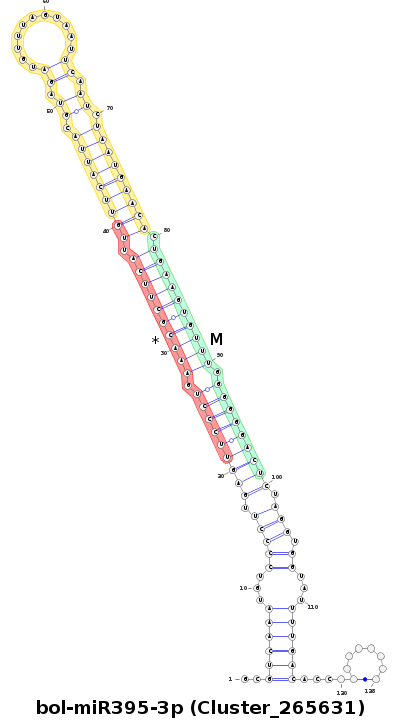

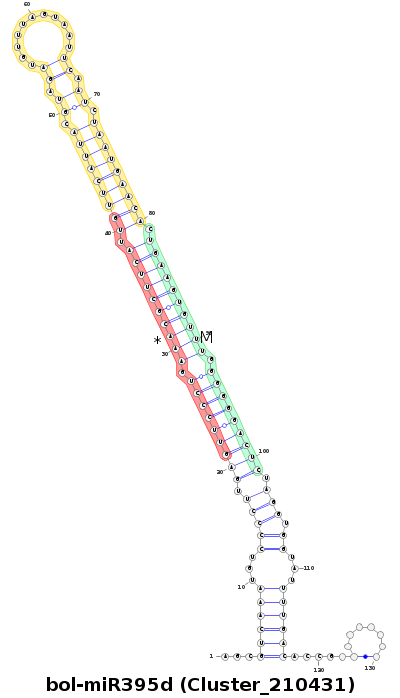

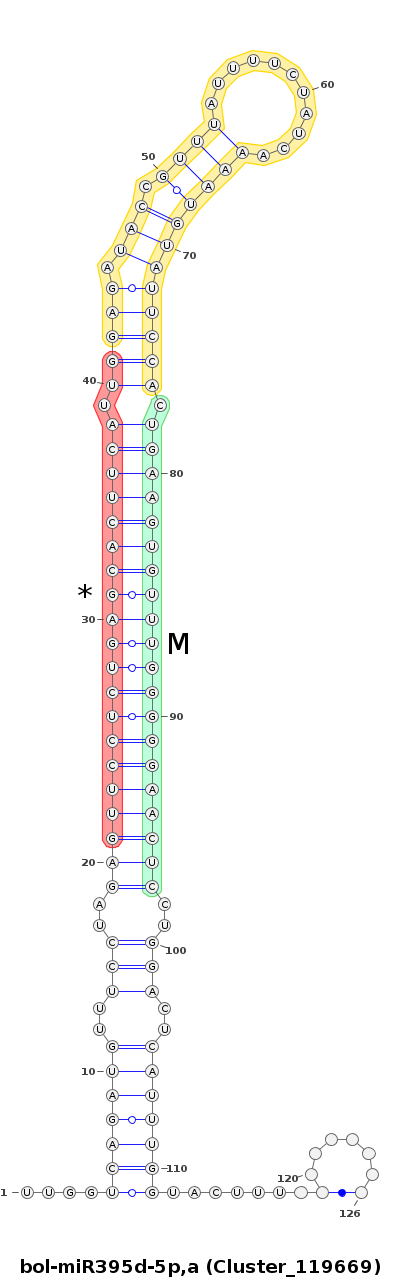

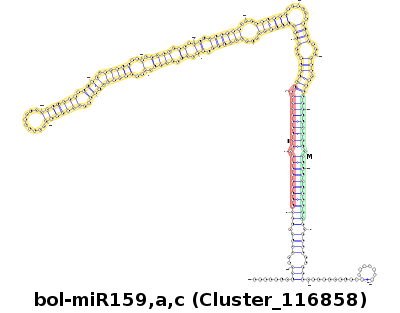

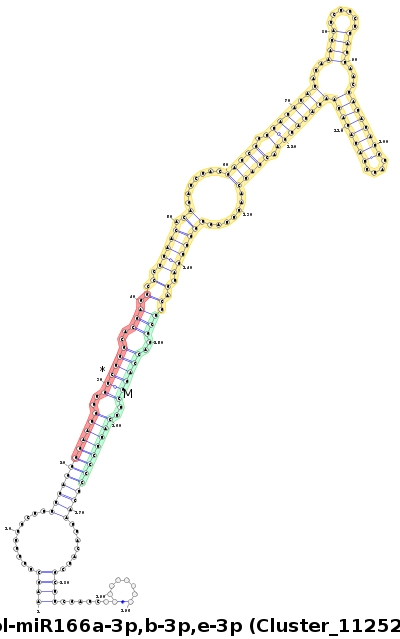

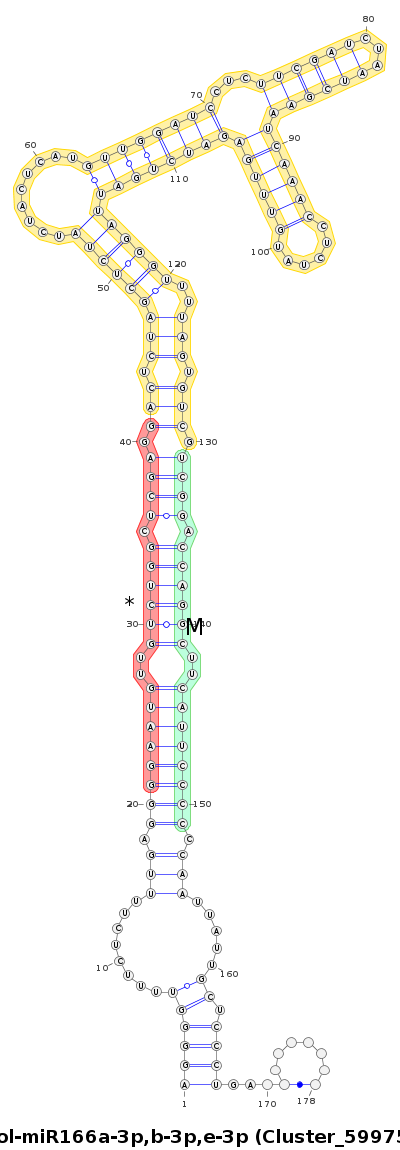

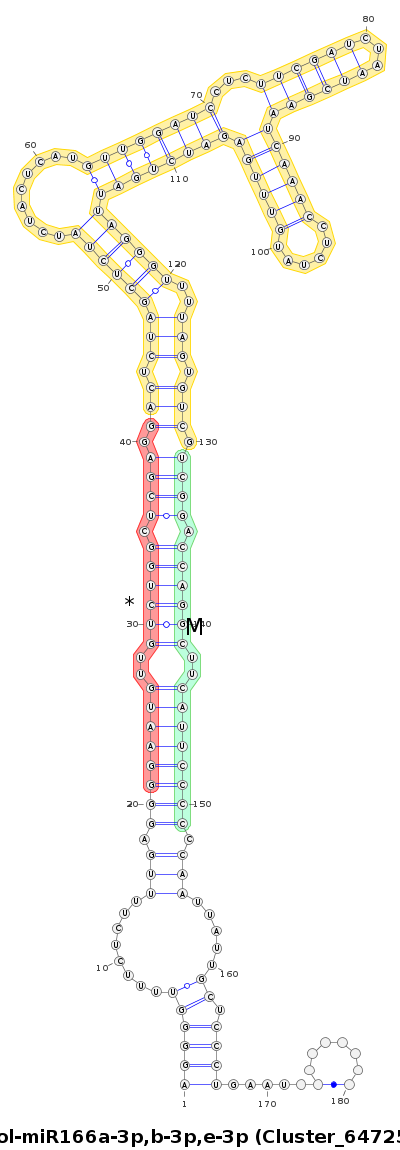

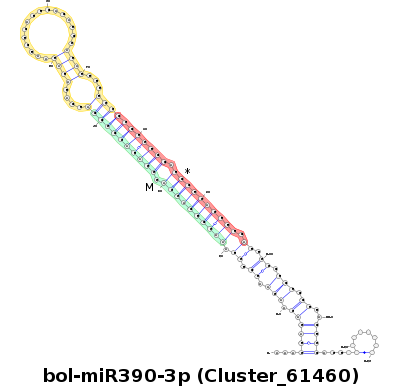

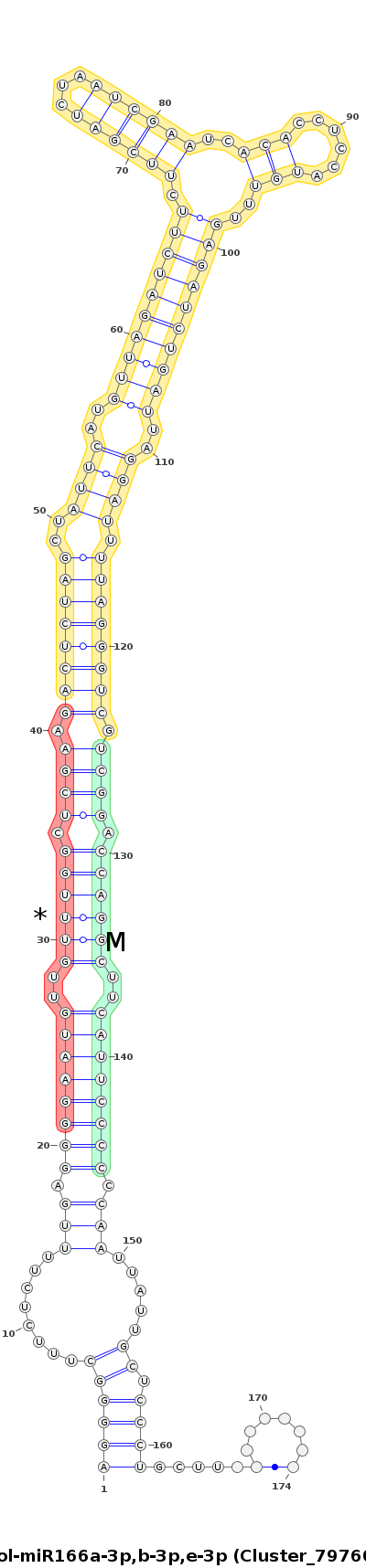

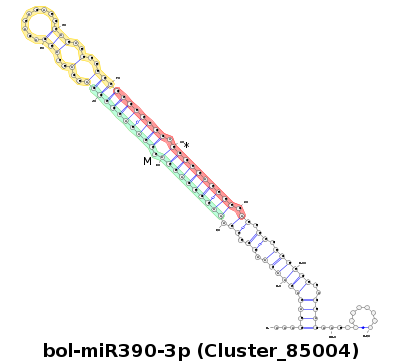

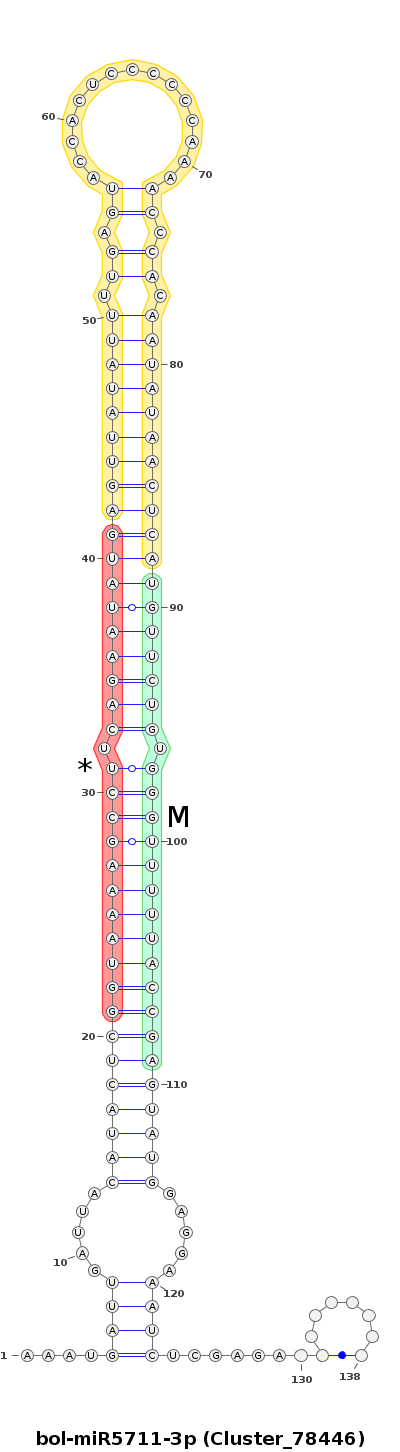

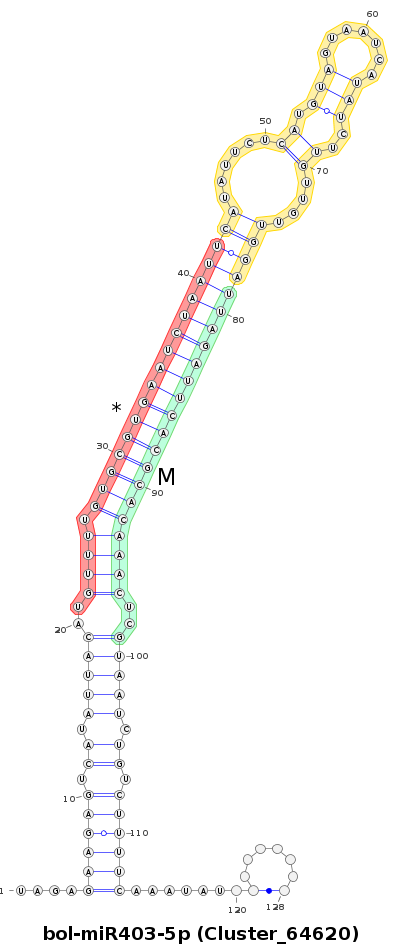

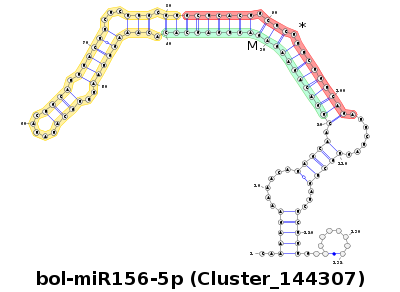

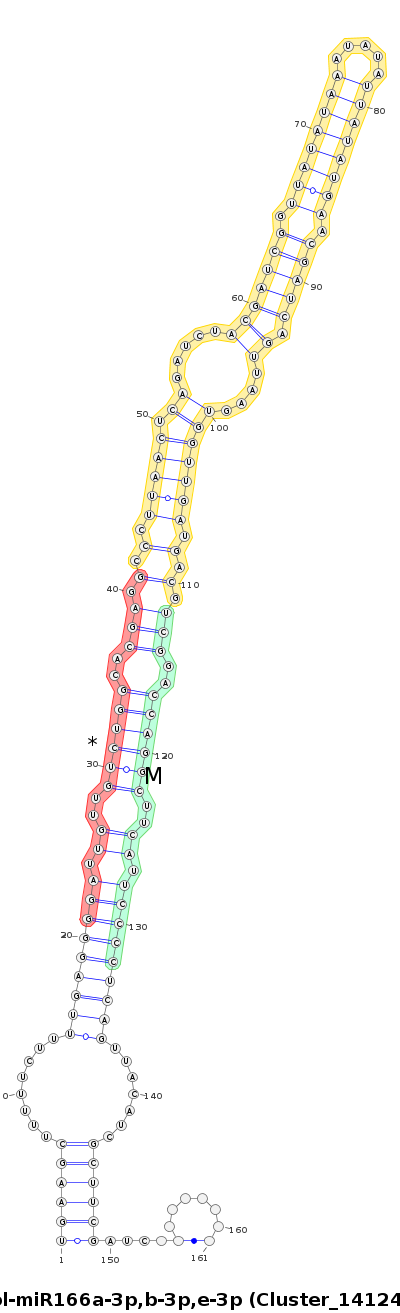

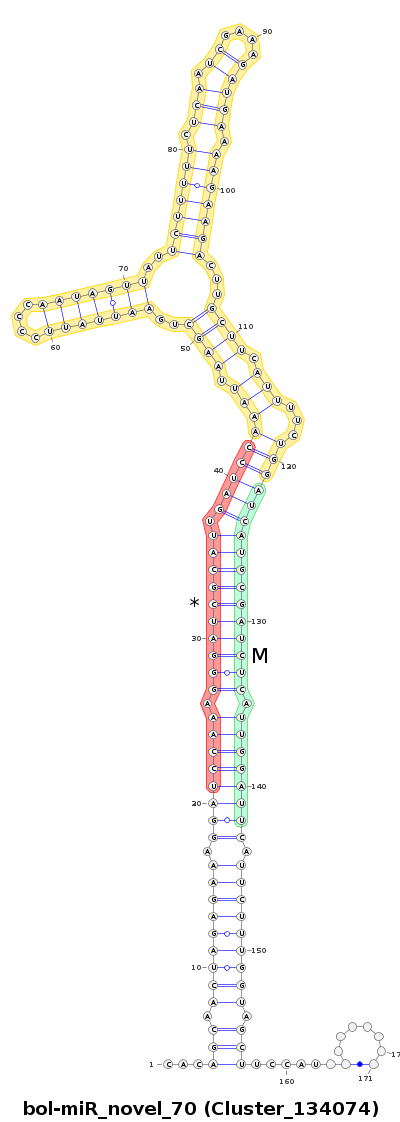

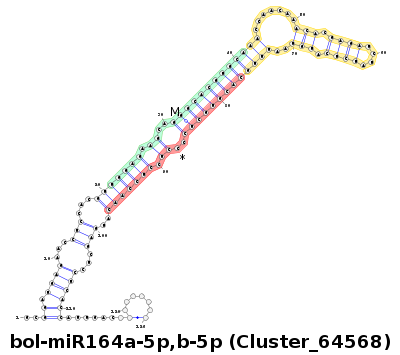

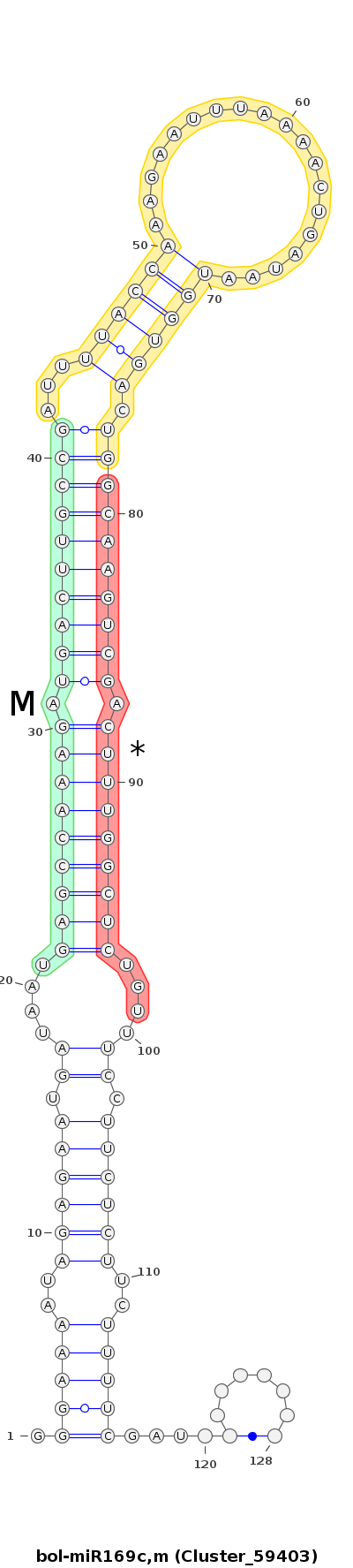

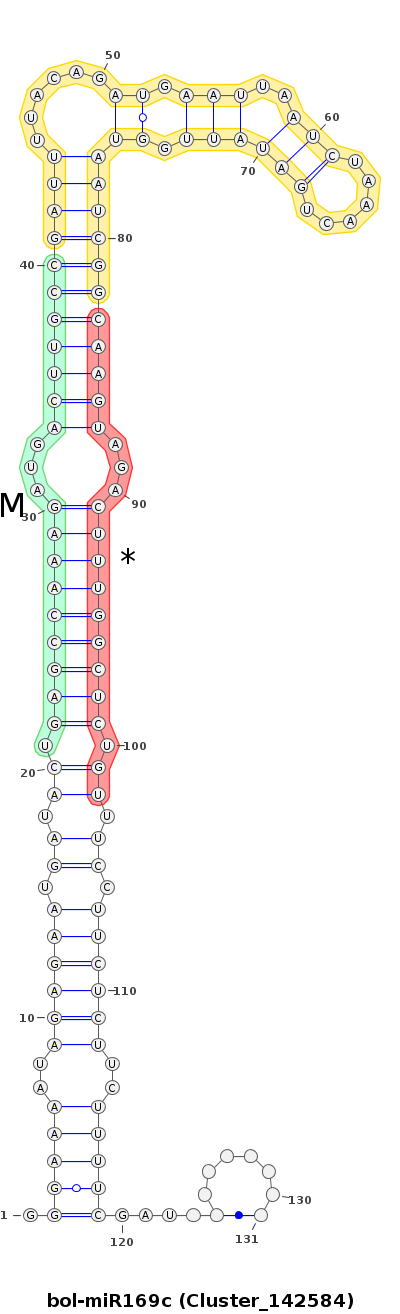

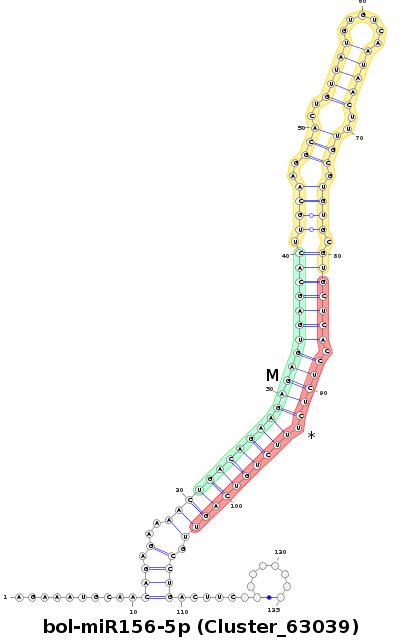

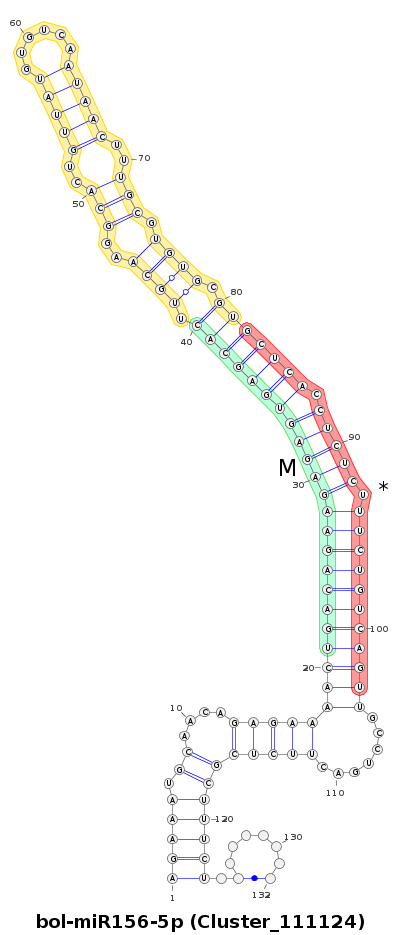

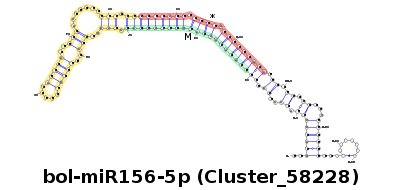

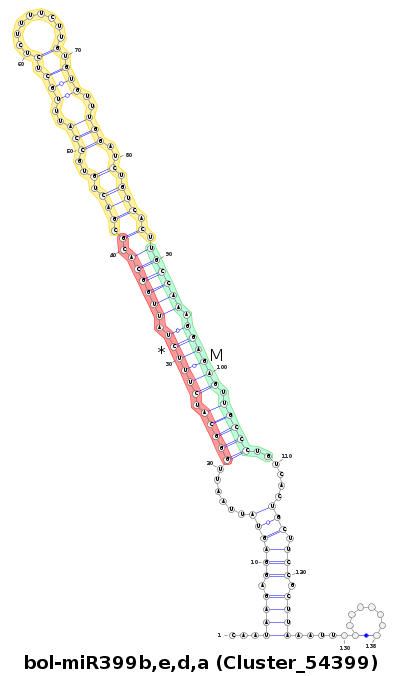

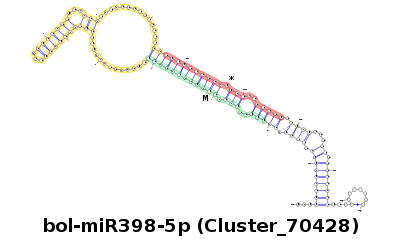

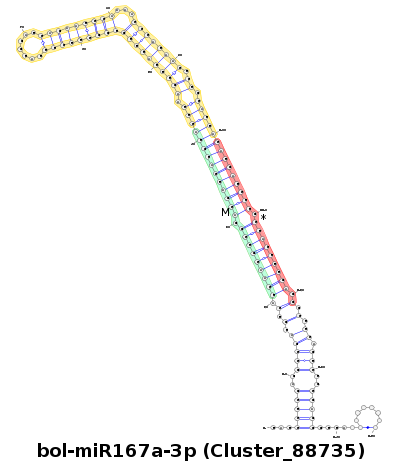

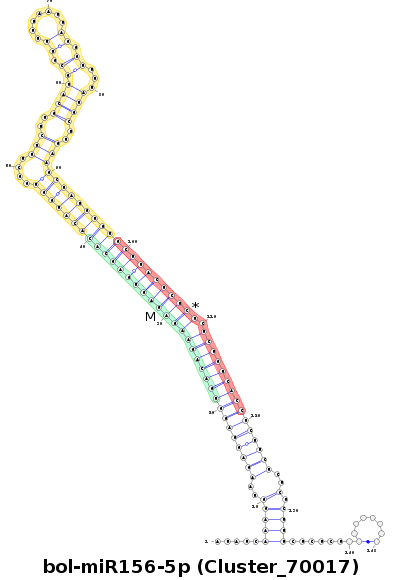

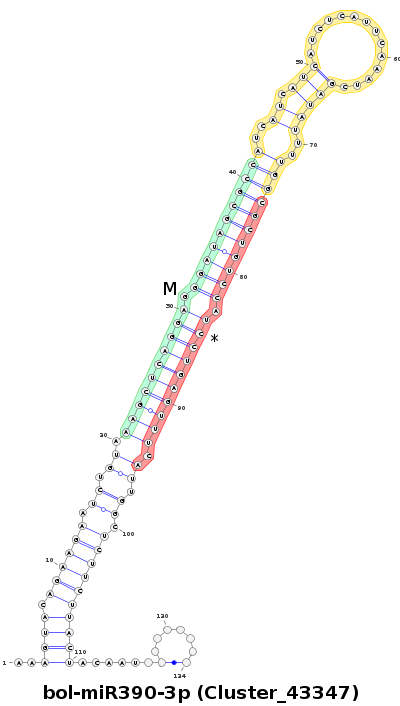

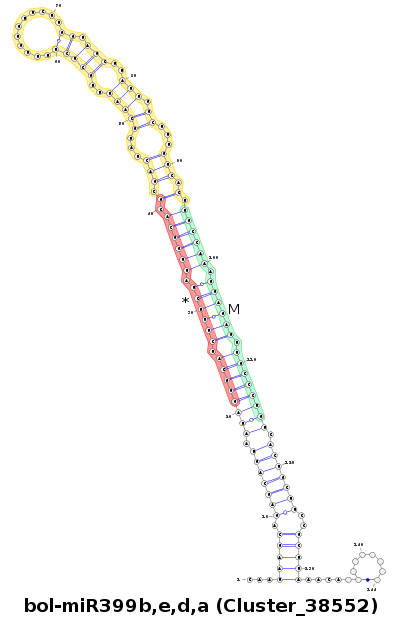

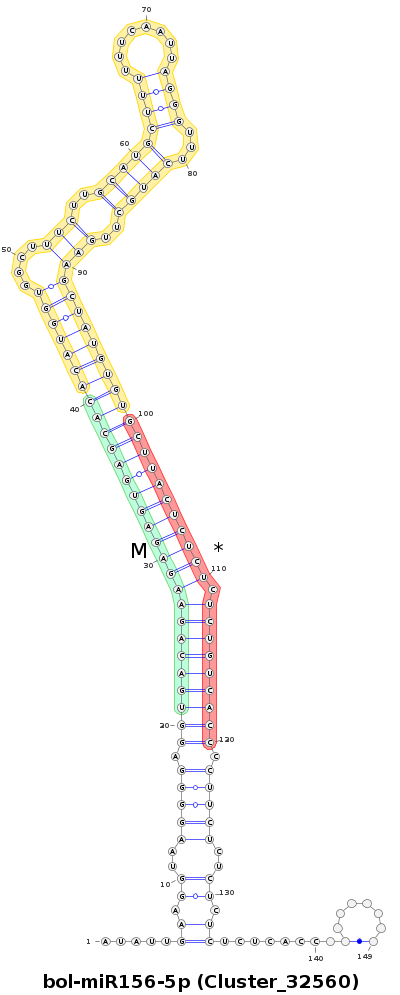

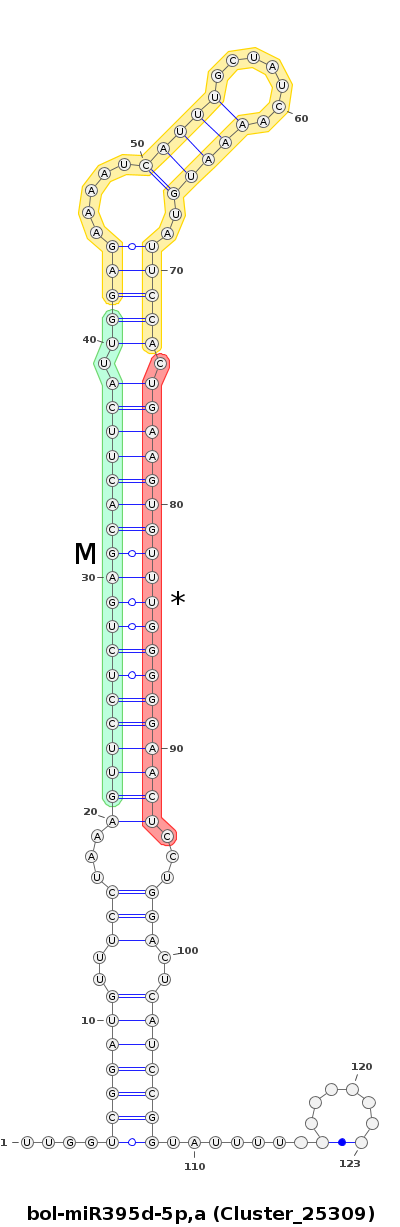

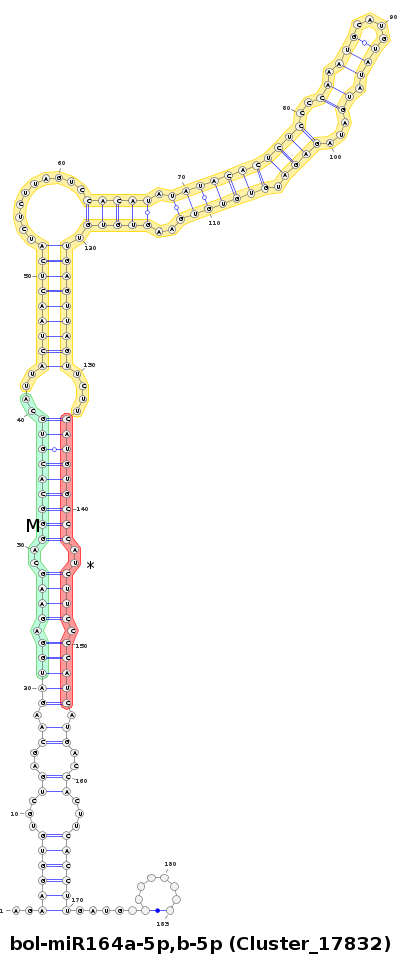

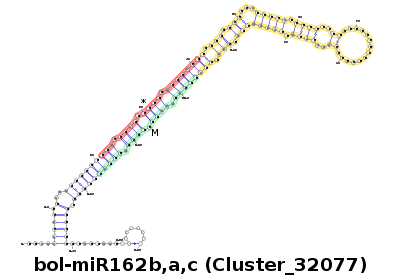

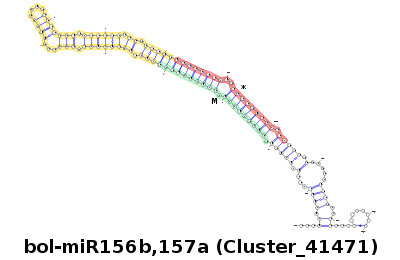

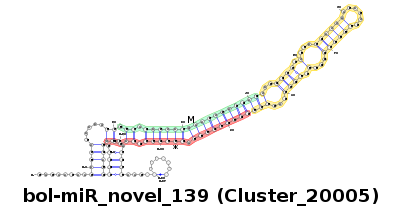

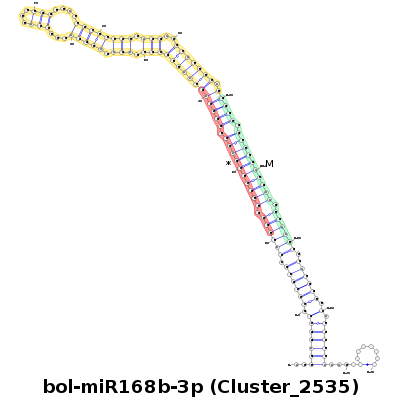

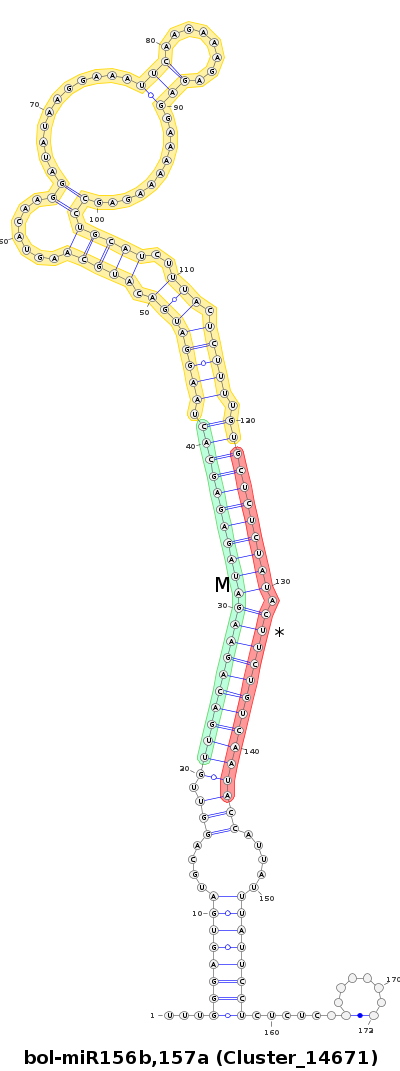


**The green peach aphid gut contains host plant microRNAs identified by comprehensive annotation of *Brassica oleracea* small RNA data**

Max C. Thompson, Honglin Feng, Stefan Wuchty, and Alex C. C. Wilson

Secondary structures for all *B. oleracea* precursors with miRNAs found in aphid gut. Green, red, and yellow highlight the mature miRNA sequence, star miRNA sequence, and loop region respectively.
